# From intravascular imaging to adaptive vascular care: intelligent photonics and digital twins in panvascular disease

**DOI:** 10.1038/s41377-026-02410-6

**Published:** 2026-08-04

**Authors:** Lingsen You, Jiaxin Yao, Yaoqing Qiu, Yu Wang, Yunlu Sun, Rongjun Zhang, Li Shen, Junbo Ge

**Affiliations:** 1 Department of Cardiology, Shanghai Institute of Cardiovascular Diseases, Zhongshan Hospital, Fudan University, Shanghai, 200032 China; 2 National Clinical Research Center for Interventional Medicine, 200032 Shanghai, China; 3 Oriental Panvascular Devices Innovation College, University of Shanghai for Science and Technology (USST), Shanghai, 200093 China; 4 State Key Laboratory of Cardiovascular Diseases, Zhongshan Hospital, Fudan University, 200032 Shanghai, China; 5 NHC Key Laboratory of Ischemic Heart Diseases, 200032 Shanghai, China; 6 Department of Cardiology, Shidong Hospital, Yangpu District, 200438 Shanghai, China; 7 Department of Optical Science and Engineering, College of Future Information Technology, Fudan University, 200433 Shanghai, China

**Keywords:** Biophotonics, Fibre optics and optical communications

## Abstract

Panvascular diseases (PVDs) stand as the leading cause of global mortality, necessitating a paradigm shift from local anatomical repair to the systemic restoration of vascular homeostasis. While intravascular optical imaging has revolutionized diagnosis, it remains a passive observation tool, restricted by “physical bottlenecks” in resolution and “cognitive bottlenecks” in interpretation. To address these challenges, we frame our analysis around “Suitcordance”, a concept aiming to capture the dynamic state of matching between interventional devices and the vascular microenvironment. In this review, we use Suitcordance as a working analytical framework to represent such a clinically-targeted, integrated perspective, and to organize the evidence on intravascular optical imaging and its integration with artificial intelligence. First, we summarize recent advances in intravascular imaging modalities, including micro-OCT, hybrid systems, and emerging detection technologies. Second, we review how AI-based image analysis and image-derived digital twin models are being applied to interpret these data and to support procedural decision-making. On this basis, we discuss how such tools may contribute to a more individualized assessment of device-vessel matching in panvascular disease.

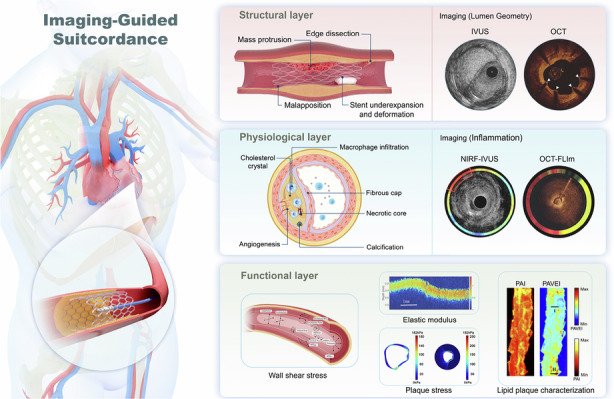

## Abstract (web summary)

AI-empowered intravascular optical imaging is evolving from “seeing clearly” to “seeing thoroughly.” A three-tiered framework, structural, functional, physiological, advances Suitcordance: device–vessel matching guiding individualized therapy toward sustained panvascular homeostasis.

## Introduction

Panvascular diseases (PVDs) encompass a series of vascular conditions characterized by the common pathological features of atherosclerosis, primarily affecting vital organs such as the heart, brain, kidneys, and limbs^1^. Driven by aging populations and lifestyle changes, the incidence of PVDs is on the rise^2,3^, posing an increasing threat to human health. As a major component of PVDs, cardiovascular diseases (CVDs) have become the leading cause of death and a primary contributor to disability globally. According to the World Heart Federation’s World Heart Report 2023, CVDs affect over 500 million people worldwide, with the annual death toll continuously increasing^4,5^.

The pathogenesis of PVDs is highly correlated with the disruption of the vascular homeostatic triad: (1) mechanical ecological balance (e.g., vascular stress, hemodynamics), (2) cellular ecological balance (e.g., endothelial cells, smooth muscle cells, immune cells), and (3) physicochemical-immune ecological balance (e.g., temperature, pH, lipids, lactate, immune-related factors). Under normal physiological conditions, these three balances exist in a state of dynamic equilibrium. In PVD, this equilibrium is broken, leading to vascular dysfunction, endothelial injury, and a cascade of homologous diseases, including atherosclerosis^6,7^.

Currently, interventional therapy serves as the primary strategy for re-establishing the tripartite ecological balance within the panvascular system^8^. To quantify this reconstruction, we frame our assessment around the concept of “Suitcordance,” which is defined as the capacity of a device to achieve both short-term suitability and long-term concordance with the intravascular environment. Short-term suitability addresses immediate mechanical matching (e.g., stent expansion and apposition), while long-term concordance captures cellular and immunological integration over time. Existing metrics each reflect only one dimension—compliance mismatch/radial-strength matching only captures the mechanical axis, biocompatibility/hemocompatibility primarily concern the biological interface, vascular healing mainly captures pathological progression along the temporal axis. Each captures one axis, yet no current concept integrates the full spectrum of homeostatic dimensions—mechanical, cellular, and immunological—across both short- and long-term scales within the context of panvascular medicine. We therefore adopt “Suitcordance” as an analytical framework to denote the unified three‑axis coordination of mechanical suitability and biological concordance across the full temporal continuum. This perspective aligns with the contemporary principle of panvascular therapy, which prioritizes sustained homeostasis beyond acute procedural success^7,9,10^.

In the era of Generative AI and intelligent medicine, achieving this state extends beyond the material properties of the device itself; it is intrinsically governed by surgical precision and computational guidance. From this integrated, clinically targeted perspective on Suitcordance, we consider two complementary components. On the imaging side, intravascular optical modalities (e.g., OCT, NIRS, photoacoustic probes) provide high-resolution, multi-dimensional information about the lumen and vessel wall. On the computational side, AI-based image analysis and image-derived digital twin models are being developed to interpret these data and to inform procedural decision-making. We use “Suitcordance” in this review to describe, in qualitative terms, the state in which imaging information and computational inference jointly support a better match between the interventional device and the local vascular environment.

Historically, techniques such as ultrasound, computed tomography (CT), magnetic resonance imaging, and digital subtraction angiography (DSA) constituted the primary modalities for PVD diagnosis^11–15^. These external modalities are constrained by fundamental physical trade-offs: limited spatial resolution, limited ability to phenotype atherosclerotic plaques at the molecular level, and limited functional hemodynamic information. Consequently, they do not provide the lumen-level detail required for the device-vessel matching considered in this review. In contrast, intravascular imaging (IVI), by virtue of its ability to be placed directly within the vessel lumen, offers a pathway to overcome these limitations, providing the high-fidelity, real-time data required. Such advances position IVI to evolve into an integrated optical imaging platform that can help close the loop between diagnosis and treatment of PVDs. Together, these capabilities make IVI a key enabling technology for operationalizing the Suitcordance framework, and underscore the need for its continued development.

**Fig. 1:**
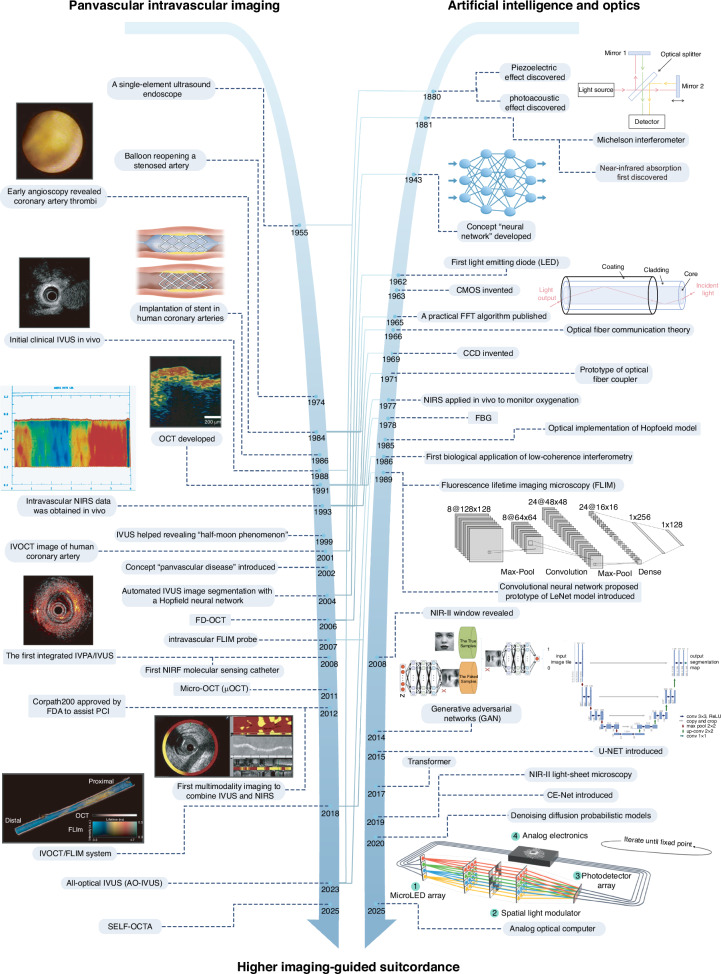
Timeline showing representative milestones in panvascular intravascular imaging alongside developments in optics and AI, illustrating the gradual integration of these fields. IVOCT: intravascular optical coherence tomography. FD-OCT: frequency-domain OCT. IVPA: intravascular photoacoustic. SELF-OCTA: spectrally extended line field optical coherence tomography angiography. CMOS: complementary metal-oxide-semiconductor. CCD: charge-coupled device. FBG: Fiber Bragg Grating. (reproduced with permission from ref. ^74^ ©2015 Springer Nature, ref. ^325^ ©1991 AAAS, ref. ^326^ ©2014 Elsevier, ref. ^327^ ©1993 ACS, ref. ^312^ ©2020 Kuku et al., licensed under CC BY 4.0, ref. ^280^ ©2018 Lee et al., licensed under CC BY 4.0, ref. ^328^ ©2019 LeNail et al., licensed under CC BY 4.0, ref. ^329^ ©2019 Luo et al., licensed under CC BY 3.0, ref. ^330^ ©2015 Springer Nature, ref. ^331^ ©2025 Kalinin et al., licensed under CC BY 4.0.)

With subsequent breakthroughs in photonics, micro-fabrication, and computational augmentation, IVI has evolved progressively. Technologies such as Optical Coherence Tomography (OCT), Intravascular Ultrasound (IVUS), and Near-Infrared Spectroscopy (NIRS) emerged, providing cross-sectional imaging with micrometer-scale resolution and chemical composition profiling to guide panvascular theranostics^16,17^ (Fig. 1). These advances enable high-precision structural and functional imaging in complex vascular environments, supporting improved device-vessel matching under imaging guidance (referred to in this review as Imaging-Guided Suitcordance). In this review, Imaging-Guided Suitcordance is used as a conceptual lens to describe how IVI may support device–vessel matching across the pre-, intra-, and post-procedural continuum. Nevertheless, widespread adoption faces physical and operational bottlenecks: high dependency on operator interpretation, latency in real-time volumetric reconstruction, and procedural risks in complex anatomies^17–19^.

**Fig. 2:**
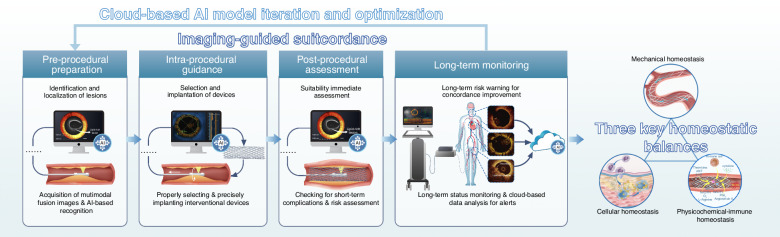
Conceptual overview of Imaging-Guided Suitcordance. Intravascular optical imaging and AI-based analysis may support clinical decision-making across the interventional workflow, from pre-procedural planning and intra-procedural navigation to post-procedural evaluation and longitudinal follow-up. Iterative model updating has also been explored to link these phases, in order to support more individualized assessment of the three homeostatic balances discussed in this review. (reproduced with permission from ref. ^104^ ©2020 Ono et al., ref. ^332^ ©2021 Yang et al., ref. ^333^ ©2023 Cai et al., all licensed under CC BY 4.0; ref. ^334^ ©2013 Oxford University Press, ref. ^335^ ©2008 Oxford University Press, ref. ^106^ ©2013 Optica Publishing Group.)

Against this background, AI has been increasingly explored as a complementary tool. Recent work has combined AI with photonic imaging technologies (Computational Photonics) to help address some of these limitations. On the optics side, AI-based methods have been used to extend the information obtainable from intravascular sensors and to support newer techniques such as Optical Coherence Elastography (OCE) and Near-Infrared Fluorescence (NIRF)^20,21^ to assess relevant lumen- and wall-level changes with improved sensitivity. On the computational side, AI-based analysis is shifting the role of IVI from predominantly manual image interpretation toward partially automated, data-driven analysis. Representative applications include learning-based super-resolution reconstruction, automated recognition of pathological features, and outcome prediction using large medical models. In combination, these tools may contribute to decision support (Fig. 2) across pre-procedural planning, intra-procedural guidance and post-procedural assessment, and so help to assess device-vessel matching more systematically.

To elucidate this paradigm shift, this review is structured as a strategic roadmap moving from passive observation to active homeostasis. We begin by dissecting the physical and cognitive bottlenecks of current intravascular modalities, establishing the critical starting point. We then review recent progress in IVI technologies, including higher-resolution optical approaches and multimodal combinations, and summarize how AI-based analysis and image-derived digital twin models are being used to interpret these data. On this basis, we discuss how such tools may support a more individualized assessment of device-vessel matching in PVD.

### Methods

This is a narrative review focusing on the integration of artificial intelligence (AI) with IVI. To identify relevant contributions, we conducted a literature search in PubMed and Web of Science as the primary databases, with Google Scholar used as a supplementary search tool to identify additional studies that might not have been indexed in the primary databases. Studies related to IVI, AI, intelligent photonics and digital twins were retrieved. The following keywords and their derivatives in different combinations were used: IVI, intravascular OCT, IVUS, NIRS, Angioscopy, multimodal imaging, panvascular medicine, image processing, image reconstruction, segmentation, lesion recognition, interventional robot, AI, deep learning, and theranostics. AI-related research mainly included studies published after 2020. Pre-2020 studies were primarily focused on clinical research, or were included when necessary to explain foundational principles or to provide historical context. Only peer-reviewed papers were included; abstracts, preprints (e.g., arXiv and medRxiv) and case reports were excluded.

The review is organized according to a pre-/intra-/post-procedural workflow to reflect the clinical continuum: pre-procedural planning and lesion assessment, intra-procedural guidance and real-time decision support, and post-procedural evaluation and follow-up. This structure allows a clinically oriented discussion of AI and optical applications across different stages of vascular intervention, from initial diagnosis to long-term management.

## Principles of panvascular intravascular imaging technologies

This section focuses on four IVI modalities, OCT, IVUS, NIRS and Angioscopy, because these are the catheter-based techniques that have been most extensively used in clinical and preclinical panvascular interventional practice and for which the largest body of AI-related literature is currently available. OCT and IVUS provide complementary structural information about the lumen and vessel wall, NIRS adds lipid-compositional information, and Angioscopy contributes direct surface visualization. Other intravascular approaches (e.g., intravascular photoacoustic imaging and fluorescence-based techniques) are largely at earlier stages of clinical translation. Moreover, most of these emerging approaches are built upon the principles of the four core technologies, and their potential is discussed in section “Pre-procedural assessment and technical preparation” in the context of multimodal or next generation systems rather than as established clinical tools. Accordingly, this review concentrates on OCT, IVUS, NIRS, and Angioscopy. This scoping is intended to keep section “Principles of panvascular IVI technologies” aligned with the main body of AI-integrated evidence reviewed in the subsequent sections.

### Optical coherence tomography (OCT)

OCT is a minimally invasive, near-infrared light-based interferometric imaging modality that provides micron-scale resolution (~10–20 μm) for superficial vascular microstructures^22^.

**Fig. 3:**
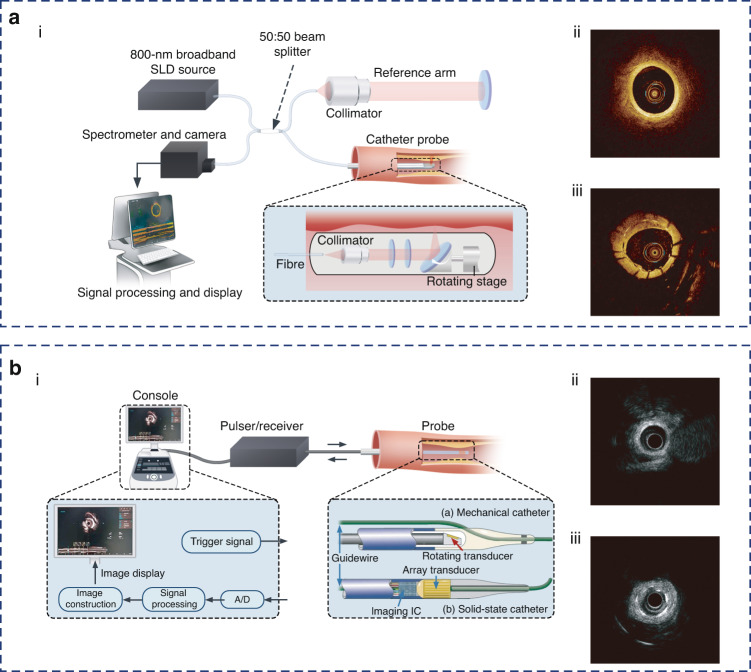
Imaging results and structural visualization from OCT and IVUS. **a** (i) Schematic of OCT principles. (ii) Representative intravascular OCT imaging scene (B-scan). (iii) Representative intravascular OCT imaging scene of a segment with stent. **b** (i) Schematic of IVUS principles. IC: integrated circuit. A/D: analog-to-digital conversion. Image provided courtesy of Boston Scientific. ©2026 Boston Scientific Corporation or its affiliates. All rights reserved. (ii) Representative intravascular IVUS imaging scene. (iii) Representative intravascular IVUS imaging scene of a segment with stent

OCT imaging, whose basic structure is shown in Fig. 3a(i), is based on Michelson interferometry. The system uses a superluminescent diode (SLD) or a broadly tunable laser with narrow linewidths as its light source. In Fig. 3a(i), the broadband light emitted by the SLD is split into two independent paths by a beam splitter. The sample arm beam scans the microstructure of the vascular tissue, while the reference arm beam undergoes phase modulation via an adjustable path delay mechanism. Backscattered light from the vessel wall interferes with the reference beam when the optical path difference lies within the coherence length, and the resulting interference signal is captured by a photodetector. Axial reflectivity profiles (A-scans) are reconstructed from this signal and, combined with high-speed longitudinal scanning and lateral encoding, synthesized into cross-sectional (B-scan) images (Fig. 3a(ii, iii)). These can be further reconstructed into three-dimensional volumetric images^22–24^.

OCT’s high spatial resolution enables detailed imaging of superficial luminal and vessel wall microstructures, including:

- Differentiation of fibrous plaques (homogeneous, high signal), lipid-rich plaques (low signal, poorly delineated), and calcified plaques (low signal with sharply defined borders)^25^.
- Identification of fibrous caps (FC), measurement of FC thickness, identification of thin-cap fibroatheroma (cap thickness <65 μm) and high-risk plaques^26,27^.
- Identification of macrophages (high-intensity, confluent signals) and neovascularization (small, signal-poor tubular or punctate structures, i.e., microchannels)^28,29^.
- Identification of thrombus with the ability to distinguish red thrombus from white thrombus^30^.
- Measurement of calcium arc, thickness, length, area, and volume, as well as differentiation between superficial and deep calcium deposits^31^.
- Identification of uncovered struts, neoatherosclerosis versus neointima^28^.
- High accuracy in detecting stent underexpansion, malapposition, and edge dissection compared with IVUS^28,32^.

OCT provides accurate, high-resolution visualization of culprit lesion morphology, enabling optimized stent implantation and reduced stent-related complications, including^25,33^:

- Precise measurement of lesion length and lumen diameter to optimize balloon and stent sizing.
- Assess plaque morphology and accurate imaging of calcium thickness. For heavily calcified lesions, consider plaque modification techniques. Avoid stent deployment in lipid-rich regions.
- Detect stent malapposition, underexpansion, edge dissection, and superficial calcium fracture, guiding post-dilation or additional stenting if needed.

Axial resolution of OCT is governed by the source coherence length and thus scales inversely with the spectral bandwidth, typically reaching 10–15 µm in clinical IVOCT systems^24,34^.

OCT is widely used in coronary arteries, where its high resolution supports detailed assessment of lesion morphology and identification of the neointimal proliferation responsible for in-stent restenosis, making it particularly suitable for culprit-lesion evaluation in acute coronary syndrome (ACS) and stent failure^31^. However, its imaging performance is bounded by the wavelength and bandwidth of the light source, and tissue scattering and absorption limit the penetration depth, leaving deep vessel-wall structures difficult to assess. Moreover, strong near-infrared scattering by erythrocytes makes imaging critically dependent on effective blood clearance, which in turn requires contrast-agent flushing. In larger vessels, particularly the left main, and in aorto-ostial lesions, the incidence of signal dropout and fold-over artifacts rises appreciably, impeding image interpretation and clinical adoption^35,36^. Inadequate blood clearance in extremely narrow or tortuous vessels (e.g., cerebral arteries), together with the relatively large integrated probe size, further constrains its use in neurovascular applications^37^.

In recent years, the automated recognition and quantification of coronary plaques using OCT has remained a major research focus, and continually optimized catheter designs together with FD-OCT technology are broadening the boundaries of practical application^37^. For example, micro-OCT (µOCT) has pushed the axial resolution down to ~1–2 µm, enabling the identification of finer structures such as endothelial cells^38^. Combining OCT with other intravascular modalities (e.g., IVUS and NIRS) and deriving OCT-based FFR provides a more comprehensive assessment of coronary plaque and post-stent haemodynamics^28^. In parallel, several biocompatible flushing media are being evaluated in clinical practice as alternatives to iodinated contrast agents, with the goal of extending OCT to patients at high risk of contrast-induced nephropathy^39,40^.

From the Suitcordance perspective, the principal value of OCT lies in its near-cellular characterization of the lumen-side device–vessel interface, which is decisive for short-term suitability (e.g., strut apposition, edge dissection). Its limited tissue penetration (~1–3 mm), however, intrinsically constrains its ability to interrogate deep plaque burden and outward remodeling, and therefore restricts OCT-only evaluation of the long-term concordance dimension of Suitcordance, which depends on vessel-wall biology beyond the superficial layer.

### Intravascular ultrasound (IVUS)

IVUS is an invasive imaging technology that employs a miniaturized catheter-based ultrasound transducer to interrogate the vessel lumen.

A standard IVUS system comprises three core components: a catheter housing a miniature single-element or phased-array transducer at its distal tip, an imaging console integrating the hardware and software for signal acquisition and reconstruction, and a motorized pullback device ensuring automated, uniform catheter withdrawal (Fig. 3b(i)). Once positioned at the target site, the transducer emits high-frequency ultrasound waves (typically 20–60 MHz) that penetrate vascular tissue. Acoustic reflections arising from differing acoustic impedances at tissue interfaces are detected by the transducer and converted into electrical signals, which are subsequently processed by the back-end system to generate grayscale cross-sectional images^41^ (Fig. 3b(ii, iii)).

IVUS transducers are classified into two primary schemes, mechanical/rotational structures and solid-state phased arrays (Fig. 3b(i))^41^. The mechanical/rotational architecture allows for significant miniaturization, facilitating navigation through stenotic lesions^41,42^. However, in tortuous vessels and at acute arterial bends, mechanical binding of the drive cable can cause non-uniform rotational distortion (NURD), producing a wedge-shaped smeared image that hinders morphological analysis^43^. During rotation, small bubbles between the catheter sheath and transducer may also disrupt ultrasound transmission, reducing image quality, which can be mitigated by saline flushing^42,44^. By contrast, solid-state arrays eliminate rotational artifacts and provide more stable imaging, but may exhibit ring-down artifacts due to near-field acoustic saturation and typically have larger tip profiles^41,45^.

IVUS’s lower spatial resolution (~100–300 μm) limits microstructural detail, but compared with OCT (~1–3 mm penetration), it offers greater tissue penetration (4–10 mm) and allows visualization of the full vessel wall, providing information on^28,43^:

- EEM area and lumen area.
- Plaque area (EEM area − lumen area) and Plaque burden ((EEM area − lumen area)/EEM area).
- Calcium arc, length, and distribution (superficial/deep), the thickness of the calcium cannot be determined.
- In vivo assessment of vascular remodeling.
- Recognize thrombus, tissue protrusion, malapposition, underexpansion and edge dissection.

In most stenting procedures, IVUS and OCT are equally safe and effective, with similar short- and long-term outcomes^46,47^. However, IVUS is preferred in certain scenarios where blood clearance or contrast administration is problematic, including^31,48^:

- Suspected spontaneous coronary artery dissection
- Aorto-ostial lesions
- LMCA PCI
- Chronic total occlusion
- Chronic renal insufficiency
- Long lesions and large vessels and deep calcium.

The resolution and penetration depth of IVUS imaging are constrained by acoustic transducer frequency and material properties. While higher acoustic frequencies enhance axial resolution, this comes at the cost of the reduced penetration depth and increased scattering^49^. Conventional piezoelectric lead zirconate titanate (PZT) transducers, despite their ubiquity, are limited by narrow bandwidths and a high acoustic impedance mismatch with human tissues, which hinders their miniaturization and high-density integration. These factors collectively constrain the spatial resolution of high-frequency IVUS^45,49^. In recent years, high-frequency IVUS based on (1-*x*)Pb(Mg_1/3_Nb_2/3_)O_3_-*x*PbTiO_3_ (PMN-PT) and Pb(In_1/2_Nb_1/2_)O_3_-Pb(Mg_1/3_Nb_2/3_)O_3_-PbTiO_3_ (PIN-PMN-PT) composite materials has been mature, typically achieving 86% bandwidth and an axial resolution as small as 43 μm at a center frequency of 41 MHz^50^. Moreover, the technology of micro-electro-mechanical systems has been driving the development of ultrasonic micro-transducers, and their convenient integration with CMOS sensors and arrays can further enhance IVUS imaging performances^45^.

Within the Suitcordance framework, this resolution–penetration trade-off positions IVUS as a complementary, rather than competing, modality to OCT: IVUS provides the deep-wall and full-thickness information required to assess long-term concordance with vessel-wall remodeling, but its lower spatial resolution (~100–300 µm) limits sensitivity to the micron-scale features (uncovered struts, fine malapposition, edge dissection) that govern short-term suitability. This explains why neither OCT alone nor IVUS alone can fully support a Suitcordance assessment, and motivates the multimodal fusion strategies discussed in Section 3.4.

With the enhancement of computational analysis capabilities, radiofrequency RF data analysis was integrated into IVUS, broadening its analytical dimensions and leading to the development of virtual histology intravascular ultrasound (VH-IVUS)^51^. VH-IVUS represents a functional extension and enhancement of conventional grayscale IVUS. It not only analyzes the amplitude signals carried by the acoustic waves but also collects and analyzes the underlying raw radiofrequency signals. This provides a qualitative analysis of plaque tissue composition, enabling in situ automatic tissue classification and the identification of high-risk lesions based on their composition.

### Near-infrared spectroscopy (NIRS)

NIRS, as an invasive catheter-based optical spectroscopic technique, applies near-infrared light as the pump or probe, and measures or visualizes the spectra of light reflected, scattered, or emitted by the targets (i.e., tissues or lesions here). Optical spectroscopies and relevant spectral imagings reveal plenty of chemical information, which can be implemented intravascularly by advanced integrated photonics. By analyzing the spectral signals generated from the interaction between near-infrared pump light and the vessel wall tissue, it provides information on the chemical compositions and their spatial distribution within the vessel wall.

Analyzing this chemical and spatial information is crucial in PVD. Myocardial infarction is often caused by lipid-rich coronary artery plaques, known as vulnerable plaques^52,53^. NIRS is highly effective in detecting the lipid core of plaques, with the extent of lipid burden expressed as the lipid core burden index (LCBI), thereby aiding clinical decision-making. This is particularly beneficial for long‑term stability within our “Suitcordance” framework: while most imaging modalities face difficulty in assessing the composition of the vessel wall, NIRS enables quantitative evaluation of lipid content and distribution, thereby providing valuable guidance for the prediction of future adverse cardiovascular events and helping to optimize long-term treatment outcomes^54–58^. However, as NIRS does not inherently provide structural information, it often needs to be combined with other IVI modalities capable of structural assessment, which in turn places higher demands on the development of multimodal fusion.

The near-infrared (NIR) spectrum corresponds to electronic and vibrational bands of molecules, thus photons within this wavelength range can induce transitions in molecular vibrational energy levels^59^. Overtones and combinations of C-H, N-H, O-H, and C=O group vibrations are responsible for the main absorption of NIR light^60–62^. Typically, NIRS quantifies lipids primarily through two characteristic peaks at 930 nm and 1210 nm^60,61^, which derive from methylene groups. In the NIR band, the absorbance of hemoglobin and water is relatively low. Therefore, NIR light can penetrate blood to identify organic substances relatively deeply in tissue with minimal interference from other components^63,64^.

**Fig. 4:**
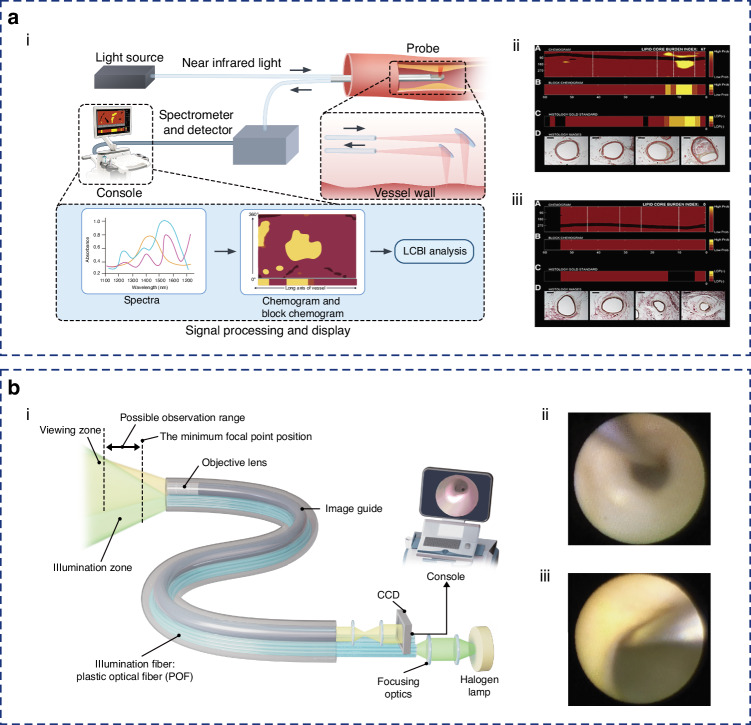
Imaging results and structural visualization from NIRS and Angioscopy. **a** (i) Schematic of NIRS principles for chemical detection. (ii, iii) Representative imaging results of NIRS, yellow in the chemogram or block chemogram indicates high probability of lipid plaques. (reprinted with permission from ref. ^56^ (©2008 Elsevier) **b** (i) Schematic of Angioscopy principles. (ii, iii) Representative Angioscopy imaging scenes demonstrating plaques with different grades^71^. (©2025 Tanita et al., licensed under CC BY 4.0)

A NIRS system consists of a rapid-exchange catheter, a pullback and rotation device, and a console. The disposable imaging catheter contains optical fibers propagating, delivering and collecting NIR light, while the console contains the laser light source, a computer for data processing, and a user interface for display^65^, as is shown in Fig. 4a(i). A spectrogram is created after the interaction between vessel wall and NIR light. Analysis of the large number of acquired NIR spectra thereby generating a chemogram that maps this chemical and spatial information (Fig. 4a(ii, iii)). Usually, LCBI, which refers to the fraction of yellow pixels obtained from the chemogram multiplied by 1000, is used to quantitatively evaluate the burden of lipid core and predict the vascular risk^58^.

Current mainstream catheters use diffuse reflectance NIRS, which analyzes the NIR light that has been scattered and absorbed within the tissue before being collected by the catheter, enabling analysis of the tissue’s internal chemical structure. Diffuse reflectance NIRS can also be used to identify various plasma components and monitor systemic and cerebral oxygenation^66^. Other spectroscopic techniques under investigation for IVI include, but are not limited to, Raman spectroscopy (e.g., spontaneous Raman and coherent Raman schemes), fluorescence spectroscopy, and magnetic spectroscopy^67^

### Angioscopy

Angioscopy is an invasive intravascular interventional imaging technique based on visible light. It uses a flexible optical fiber to introduce white light into the vessel lumen and receives the reflected light signal from the vessel wall, enabling real-time color visualization of the endoluminal surface. Different tissues have different optical properties and thus exhibit different colors and brightness when illuminated by visible light. Under Angioscopy, atherosclerotic plaques can be classified as white or yellow, while coronary thrombi can be categorized as white, red, or mixed^68,69^. Different colors can be associated with different pathological features, thus assisting diagnosis^70,71^.

Angioscopy provides high-resolution three-dimensional color images with a resolution below 10 μm (Fig. 4b(ii, iii))^72,73^, allowing for direct observation of the surface color and morphology of atherosclerotic plaques, neointima, stent struts, and thrombi. Owing to its visible-light waveband and high resolution, it has a unique advantage in thrombus detection^68,74,75^, thereby providing an important complement to the previously discussed IVI techniques.

Angioscopy plays an important role in a variety of clinical settings. Its unique ability to discriminate thrombi and its extremely high resolution render it indispensable for the assessment of thrombotic lesions and post-stent evaluation. In the coronary arteries, it allows assessment of vulnerable plaques and thrombus characteristics^71,76,77^, verification of stent apposition and expansion^78,79^, and guidance of precise treatment for in-stent restenosis^80^. In the peripheral arteries, it can be employed to evaluate plaques and thrombi in the setting of peripheral arterial occlusive disease^81–83^. In the cerebrovascular territory, it facilitates preoperative evaluation of intracranial vascular stenosis and aneurysms^72,84–86^, as well as real-time intra-procedural navigation during interventional procedures.

However, limited by the low penetration of visible light, Angioscopy cannot provide depth information and therefore often serves as a complement to other techniques^73^. The use of Angioscopy requires either blood flow occlusion or flushing, resulting in a short duration for a single procedure and precluding prolonged continuous observation. The original Angioscopy catheter had a larger diameter than other IVI techniques, which hindered its application in small-vessel environments. Nevertheless, thanks to the development of MicroAngioscopy technology, it is gradually becoming applicable to a wider range of vascular settings^72^.

Figure 4b(i) shows the basic structure of Angioscopy. It primarily utilizes fiber-optic transmission and reflection imaging of visible light. The diagnostic range of the angioscope is bounded at the proximal end by the minimum working distance of the objective lens, and at the distal end by the limit set by the available illumination intensity and image-sensor sensitivity. A traditional Angioscopy system consists of four parts: a flexible fiber-optic angioscope, a light source, a flushing and balloon system, and a video monitoring and recording system. The light source transmits visible light into the vessel lumen via an optical fiber^87^. Since visible light has poor penetration in blood, a flushing system is required to clear the blood, or a balloon system is used to occlude blood flow and create a clear viewing field. Based on this, Angioscopy is classified into two primary types: non-obstructive and occlusion-type. Non-obstructive angioscopy, which maintains adequate blood flow throughout imaging, is relatively safe and therefore suitable for application in larger vessels such as the aorta^88^.

The image guide is the core component of the angioscope. It consists of thousands of regularly arranged and fused optical glass fibers that transmit the true optical image from the distal end of the catheter to the proximal camera or eyepiece. Most current systems use silica-based image guides, though recent research into plastic image guides shows promise for reducing catheter stiffness and cost^89^. Because modal dispersion (turbulent wavefronts from fiber bending and imprecise refractive index profiles) prevents a single fiber from accurately transmitting an image, Angioscopy uses a pixel-separation method, where each pixel is transmitted through a separate fiber. Resolution is limited by factors such as fiber diameter; the latest generation of catheters can contain 30,000 or more fibers^90^. With the recent rise of electronic endoscopes, some technologies now integrate a CMOS sensor into the angioscope tip, transmitting the imaging information as an electrical signal. This significantly reduces signal loss associated with fiber-optic transmission and markedly improves image clarity^91^.

In clinical practice, the selection of an appropriate IVI modality is guided by the specific requirements of the vascular bed, the nature of the lesion, and the procedural objective. The four modalities discussed, OCT, IVUS, NIRS, and Angioscopy, differ fundamentally in their imaging mechanisms, which in turn dictates their spatial resolution, tissue penetration, and ability to characterize plaque composition. To facilitate a systematic, side-by-side comparison of their Physical and Optical Parameters, technical specifications, Clinical Utility and Performance, a comprehensive summary is provided in Table 1.

**Table 1:** Comparison of four core intravascular imaging modalities

		OCT	IVUS	NIRS	Angioscopy
**I. Physical & optical parameters**	**Source/principle**	Near-infrared interferometry (800–1300 nm)	Acoustic wave reflection (20–60 MHz)	Spectral absorption (800–2500 nm)	Visible light reflection
	**Spatial resolution**	10–20μm	100μm	N/A (chemical only)	9μm
	**Penetration depth**	1–3 mm	4–10 mm	N/A (chemical only)	Minimal
**II. System specifications**	**Core device**	Michelson interferometer	Piezoelectric transducer	Spectrometer	Image guide/CMOS
	**Information output**	High-resolution cross-sectional image	Low-resolution full-wall image	Chemogram (lipid core map)	Real-time color surface view
	**Blood clearance/contrast agent**	Required (contrast)	Not required	Not required	Required (saline)
	**Invasiveness/access mode**	Minimally invasive; requiring temporary blood clearance by contrast flush	Minimally invasive; catheter-based, no flush required	Minimally invasive; typically integrated within NIRS-IVUS combo catheter	Minimally invasive, requires temporary blood-flow occlusion or saline flushing
**III. Clinical utility & performance**	**Primary target**	Microstructure & lumen	Vessel wall & plaque burden	Lipid composition	Surface color & thrombus
	**Key applications**	Calcification, thrombus, dissection, neointima, stent apposition	Plaque burden, remodeling, left main, chronic total occlusions guidance	Lipid core burden index (LCBI), vulnerable plaque	Plaque color grading, thrombus classification
	**Best-fit vascular bed**	Coronary (emerging cerebral applications) (calcified, ACS, stent)	Coronary and peripheral	Coronary and peripheral	Coronary (emerging cerebral applications) (thrombus)
	**Best-fit workflow stage**	Pre-, intra-, post-PCI	Pre-, intra-, post-PCI	Pre-/post-PCI (lipid assessment)	Pre-, intra-, post-PCI (surface evaluation)
	**Advantages**	High resolution with reasonable penetration	Deep penetration (full vessel wall) and no flush	Specific for lipid core	Direct color and morphology display with high resolution
	**Limitations**	Insufficient penetration and contrast reliance	Lower resolution and slower pullback; difficulty in image interpretation	No structural/morphological information	Limited to surface visualization; largely qualitative interpretation
	**Role in Suitcordance**	Optimizing stent implantation and reducing stent-related complications	Guiding sizing and reducing stent-related complications	Identifies lipid-rich plaques for risk assessment, predicting plaque rupture	Detecting thrombus and plaque type, indicating event risk
	**Innovation direction**	Contrast-free, multimodal fusion, AI analysis, penetration improvement	Higher resolution and frequency, AI analysis, sensor fusion	Combined structural imaging (e.g., OCT-NIRS)	Digital Angioscopy, AI-quantification, miniaturization
	**Ref**.	^22–24,32,72,285–287^	^32,41,42,45,50,72,200,288^	^58,59,62,64,66^	^72–74,87,89,90^

## Pre-procedural assessment and technical preparation

Pre-procedural assessment and technical preparation are prerequisites for a successful panvascular intervention. The core objective is to achieve an optimal match between the interventional device and the target vessel through precise, individualized imaging information. This initial matching is the foundation of improving Imaging-Guided Suitcordance. Optical technologies play a critical role in this process. High-resolution IVI can directly visualize the vessel wall structure, lesion type, and micro-features, providing precise navigation for device selection and positioning. Concurrently, by combining multimodal imaging with optically guided strategies, it is possible to establish the initial “suitability” between the vascular bed and the interventional device pre-procedurally. This ensures the precision of device deployment and lays the foundation for both short-term mechanical ecological balance and long-term concordance of the vascular microenvironment.

### Intravascular imaging and vascular bed suitability: the foundation of Suitcordance

PVDs involve multiple vascular beds with significant differences in structural characteristics, hemodynamics, and lesion types, which places highly personalized demands on IVI technologies. In interventional therapy, how to achieve the optimal suitability between the device and the vessel and to obtain more information valuable for the interventional procedures via an optical catheter (i.e., “Imaging-Guided Suitcordance”) directly impacts interventional safety, the precision of stent deployment, and the restoration of long-term vascular function and physiological balance. Therefore, pre-procedural, precise imaging assessment of different vascular beds is not only fundamental to determining device selection and operational strategy but is also a prerequisite for achieving concordance among the three balances of vascular mechanics, cellular ecology, and physicochemical immunity. This section will summarize the primary characteristics of the coronary, cerebral, peripheral, and renal arteries, as well as microvascular and venous systems, and assess the suitability of IVI technologies for each (Table 2). By integrating the advantages and limitations of optical imaging, we aim to construct a clinically-driven framework for panvascular assessment.

**Table 2:** Panvascular vascular-bed matrix

	Coronary arteries	Cerebral vasculature	Peripheral arteries	Renal arteries	Microvasculature	Venous system
**Key vascular/lesion features**	Small caliber, bifurcations, dynamic flow; lipid-rich plaques, thin-cap fibroatheroma (TCFA), thick-cap fibroatheroma, calcification, thrombus, remodeling	High tortuosity, wall fragility, small lumen; aneurysm-prone, fibrotic plaque, small-vessel disease	Large-caliber vessels; long diffuse lesions, CTO, severe calcification, diabetes-related disease	High blood flow perfusion; ostial atherosclerotic stenosis, FMD (mid-to-distal segments)	Very small caliber, thin vessel wall, high structural differentiation, and dynamic perfusion regulation; perfusion abnormalities, capillary occlusion, and microvascular thrombosis, most prominently in diabetes mellitus	Low-pressure, slow blood flow large capacitance, valve structures, thrombosis tendency; varicose veins, CVI, DVT
**Core Procedural Conflict**	Lesion heterogeneity and calcification-induced underexpansion and malapposition	Safe catheter navigation constrained by susceptibility to vessel injury in the setting of extreme tortuosity and fragility	Uncertainty in vessel sizing and diffuse disease burden causing incomplete lesion coverage	Elastic recoil and inaccurate lesion delineation causing imprecise ostial positioning	High-resolution structural assessment limited by physical accessibility and scale constraints	Imaging artifacts induced by compressibility and low-pressure flow constrain accurate thrombus characterization
**Intravascular Imaging Strategy**	OCT, IVUS, NIRS; IVUS-OCT, IVUS-NIRS, OCT-NIR(A)F, FLIm-OCT, IVPA-IVUS	OCT, IVUS, nOCT, HF-OCT	IVUS, OCT	IVUS	Limited IVI; optical/acoustic techniques (e.g., OCT-A, LSCI, PAI)	IVUS
**Device-Vessel Matching Guidance**	Accurately measure plaque extension and luminal obstruction, optimize stent expansion, ensure stent apposition, and detect early suboptimal PCI results	Define occlusion site, identify perforator vessels, perform submaximal angioplasty	Measure vessel diameter and lesion extent, perform long-segment reconstruction, modify calcified lesions, ensure complete lesion coverage	Guide precise aorto-ostial stent positioning and ensure complete ostial coverage	Functional assessment dominates	Accurately identify venous stenoses and optimize stent sizing and landing zones
**Limitations**	Complex lesion morphology, vulnerable plaques prone to rupture, heterogeneous local hemodynamics, and cardiac motion	Catheter size and flexibility constraints	Vessel size heterogeneity, long-segment lesions, and heavy calcification limit continuous imaging acquisition and assessment, additional ultrasound artifacts	Ostial location and elastic recoil impair accurate vessel sizing	High motion sensitivity, lack of absolute flow quantification; limited intravascular imaging evidence	Dynamic venous geometry, lack of standardized stenosis criteria, operator dependency, and limited clinical evidence
**Refs**	^28,111,203,289,290^	^204,213,214,229,291^	^48,292,293^	^294–296^	^297–300^	^301–303^

*CTO* chronic total occlusion, *FMD* fibromuscular dysplasia, *CVI* chronic venous insufficiency, *DVT* deep vein thrombosis, *PAI* photoacoustic imaging, *LSCI* laser speckle contrast imaging

### Photonic innovations in optical parameters to enhance Suitcordance

Beyond selecting the right modality for a given vascular bed, optimizing the core optical parameters of that modality is crucial for enhancing diagnostic precision and ultimately improving Imaging-Guided Suitcordance. By optimizing core parameters such as light source bandwidth, wavelength, polarization control, and laser power, IVI technologies, including OCT, NIRS, and IVUS, can achieve multidimensional capabilities, ranging from micron-scale structural analysis to molecular and functional assessment. From the perspective of Imaging-Guided Suitcordance, these capabilities are particularly conducive to treating the complex vascular environment as an integrated whole, thereby improving both long-term and short-term outcomes through enhanced mechanics and physiology.

The resolution and penetration depth of IVI technologies are closely related to the bandwidth and frequency of the light/sound source. Therefore, the existing imaging performance can be improved by modifying these optical parameters.

**Fig. 5:**
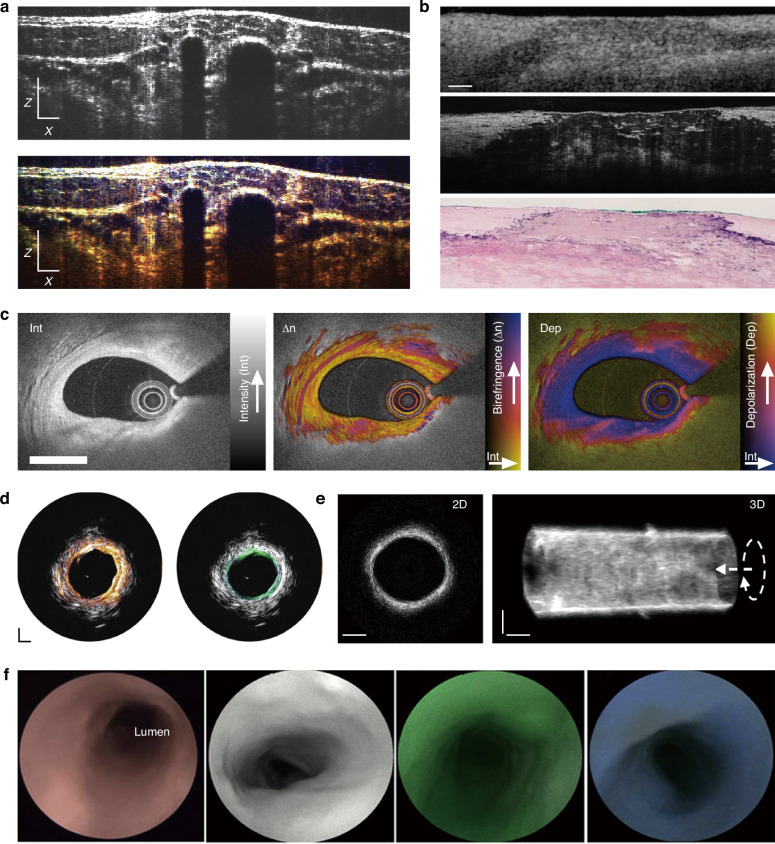
A presentation of imaging results from selected innovative technologies. **a** Conventional OCT (top) and METRiCS OCT (bottom) images. White *x* and *z* scale bars: 100 μm. (reprinted with permission from ref. ^92^ © 2011 Springer Nature) **b** Comparison between corresponding OCT (top), μOCT (middle), and histology images (bottom) of a calcium plate within the coronary artery wall. (reprinted with permission from ref. ^93^ © 2011 Springer Nature) **c** Polarization-sensitive optical frequency domain imaging signals measured in vivo in coronary artery, including intensity (Int) of the reflection signal in a conventional logarithmic gray scale, birefringence in color hue overlaid on the reflection signal (Δn), in areas of low depolarization, and depolarization in color hue overlaid on the reflection signal (Dep) indicating zones of pronounced depolarization. Scale bar: 1 mm. (reprinted with permission from ref. ^99^ © 2018 Elsevier) **d** Fused IVUS images of a human coronary artery captured by (left) 35/90-MHz multi-frequency IVUS catheter and (right) 35/120-MHz multi-frequency IVUS catheter. White: 35-MHz IVUS image, Orange: 90-MHz IVUS image, Green: 120-MHz IVUS image. Dynamic range: 50 db. Scale bar: 1 mm. (reprinted with permission from ref. ^97^ © 2015 IEEE) **e** Cross-sectional 2D image acquired by AO-IVUS (left) and 3D ultrasound data of arterial wall by rotational pullback scan of AO-IVUS (right). Scale bars, 1 mm. (reprinted with permission from ref. ^101^ © 2023 AAAS) **f** Scanning fiber Angioscopy images of normal carotid artery with different reflectance and fluorescence. (reprinted with permission from ref. ^102^ © 2017 Springer Nature)

Molecular imaging true-color spectroscopic optical coherence tomography (METRiCS OCT) is based on spectroscopic OCT, and combines a visible-spectrum light source with a dual-window processing method, with a center wavelength of 575 nm and a bandwidth of 240 nm. Covering the visible range, it enables molecular imaging of a wide range of absorbers, including hemoglobin and various contrast agents. It demonstrates advantages in the high-sensitivity detection of low hemoglobin concentrations or minute hemoglobin changes, and provides true-color contrast and rich imaging information^92^, as shown in Fig. 5a. The Micro-OCT (μOCT) imaging system employs an ultra-broadband light source (an 800 ± 150 nm laser-generated supercontinuum) to achieve a 1 μm axial resolution (consistent with the governing formula), and uses a relatively high numerical-aperture objective lens (numerical aperture = 0.12) and an annular apodizer to achieve a lateral resolution of 2 μm. Its comparison with traditional OCT and histopathology is shown in Fig. 5b. This can effectively assess cellular ecological balance, providing a powerful tool for predicting panvascular events and guiding treatment strategies^93^. Although NIRS has fewer innovations in optical parameters, modifying the spectral band can highlight different biochemical components and alter the penetration depth. Some research has applied narrow-band imaging, which is used in endoscopy, to Angioscopy, enhancing the imaging of specific tissues. For instance, using narrow-band illumination at 415 ± 30 nm can significantly improve the contrast of the superficial capillary network^94,95^. Innovations in optical parameters, by adjusting core source parameters like bandwidth, frequency, and polarization, can significantly enhance the resolution, contrast, and functional dimensions of IVI, enabling multi-scale, multi-contrast diagnostic capabilities from macroscopic structures to the subcellular. High-definition intravascular ultrasound (HD-IVUS), by increasing the acoustic frequency (more than 80 MHz), can theoretically achieve an axial resolution of less than 20 µm, approaching that of OCT. This allows for the precise characterization of microstructures like thin-cap fibroatheromas and shows excellent potential for simplifying catheter designs in multimodal fusion by replacing OCT, as shown in Fig. 5d. This innovation is analogous to the strategy in OCT of using shorter wavelengths or broader spectral bandwidths to enhance resolution, sharing a common ground in “parameter optimization”^96,97^.

The efficacy of these parameter optimizations is fundamentally rooted in the sophisticated manipulation of light-matter interactions within the constraints of miniaturized intravascular environments. For instance, achieving stable sub-cellular resolution in μOCT necessitates not only ultra-broadband spectral coverage but also precise coherence control of the light source to mitigate phase noise and chromatic dispersion^93^. Furthermore, as probes navigate the highly heterogeneous and scattering vascular milieu, understanding the propagation characteristics of vector light fields becomes paramount. Advanced fiber designs must incorporate waveguide mode theory to minimize modal crosstalk and polarization-mode dispersion in single-fiber or multi-core architectures, ensuring the fidelity of the retrieved phase and intensity information across tortuous trajectories.

### Innovations in optical devices to optimize Suitcordance

Parameter optimization alone is insufficient to address the practical challenges of complex vascular beds. Therefore, modifications have emerged that directly alter the optical device design at the front-end. These approaches leverage functional integration, expanded informational dimensions, and novel imaging principles to achieve superior Suitcordance in panvascular interventions. Existing technologies break through original constraints by introducing new techniques and analytical dimensions to improve imaging effects and surgical applicability. For example, Polarization-Sensitive OCT (PS-OCT), which leverages the distinct birefringence and depolarization properties of various tissues, modifies the optical frequency domain imaging (OFDI) system to perform intravascular polarization-sensitive measurements and to reconstruct maps of tissue birefringence and depolarization in addition to the conventional reflection signal, as is shown in Fig. 5c. This implementation provides a new dimension for differentiating intravascular structures, such as smooth muscle cells, collagen, lipid pools, and cholesterol crystals, without compromising high-resolution structural assessment, thereby aiding in vessel wall segmentation and the identification of high-risk plaques^98,99^.

With advancements in optical technology, the photoacoustic effect has been progressively integrated into IVUS, further enhancing its imaging quality and dimensionality. In IVPA imaging, the catheter-tip probe emits short near-infrared laser pulses to irradiate the artery wall. This utilizes the photoacoustic effect to excite broadband ultrasound waves, which are then collected by an ultrasound detector for image reconstruction. This technique can acquire the chemical characteristics of vessel wall components based on the specific optical absorption properties of different tissues, enabling the identification of vulnerable plaques and achieving high-definition, high-contrast imaging^100^. In recent years, the All-Optical IVUS (AO-IVUS) imaging system has emerged. It utilizes picosecond laser pulses to excite a carbon nanotube-polymethyl methacrylate (MWCNT-PMMA) composite to generate ultrasound waves and employs a π-phase-shifted fiber Bragg grating (π-FBG) sensor as the receiver. This overcomes the low frequency, slow response, and high noise of traditional single-crystal transducers, achieving IVUS imaging with ultra-wide bandwidth and high resolution^101^ (18.6-micron axial, 124-micron lateral), as is shown in Fig. 5e. Recently, a multimodal scanning fiber endoscope was developed, which innovated upon traditional Angioscopy in its light source, scanning fiber, and computing system. It utilizes a single scanning fiber to emit red, green, and blue laser beams to illuminate the tissue, while a ring of optical fibers located in the scanner housing and around the axis collects the backscattered reflectance light and fluorescence signals. Images are reconstructed at 30 Hz, achieving high resolution and a wide field of view. This system not only provides a massive improvement in structural imaging resolution (from 8000 pixels to 200,000–250,000 pixels) but also capture the fluorescence emission of endogenous components within the arterial tissue (Fig. 5f)^102^. This achieves synchronous multimodal imaging of both morphology and molecular function, which has significant implications for accurately assessing the three ecological balances and comprehensively enhancing Imaging-Guided Suitcordance.

Structural innovations in optical catheters are a key path to overcoming the limitations of current IVI and enhancing its applicability in complex vascular environments. However, these innovative technologies often face challenges in miniaturization, material stability, system complexity, cost control, and clinical translation. In the future, by combining advanced micro/nano-fabrication techniques, novel functional materials, and intelligent control algorithms, optical catheters are expected to achieve better performance, safer, and smarter imaging within even smaller and more tortuous vessels.

### Multimodal fusion and hybrid imaging technologies

Multimodal IVI technologies have emerged to overcome the intrinsic limitations of single-modality approaches, enabling more detailed and comprehensive visualization of vascular structure and pathology. Table 3 summarizes representative multimodal imaging platforms, including their technical architectures, clinical evidence, and limitations.

**Table 3:** Multimodal intravascular techniques

	IVUS-OCT	OCT-NIRS	IVUS-NIRS	IVPA-US	OFDI-NIRF	FLIm-IVOCT	FLIm-IVUS	MS-IVTM (Tri-modality)
**Technical Composition & Architecture**	Disposable integrated catheter; proximal drive module; real-time imaging console	OCT/OFDI and NIRS consoles; dual-modal intravascular imaging catheter; data acquisition and display part	Scanning near-infrared laser; rotary pullback unit; dual-modal intravascular imaging catheter	IVUS imaging system interfaced with a pulsed laser source.	OFDI and NIRF consoles; dual-modal intravascular imaging catheter; data acquisition and display part	OCT and FLIm consoles; dual-modal intravascular imaging catheter; data acquisition and display part	IVUS motor drive unit; multimodal catheter; data acquisition; wavelength selection module	Fully integrated photoacoustic, OCT, and ultrasound imaging modalities in a single 0.9 mm miniature catheter
**Development Stage**	Early clinical validation	First-in-man studies	Commercially available	In vivo validation	Early clinical validation	First in man studies	In vivo validation	Ex vivo validation
**Representative studies**	Sheth et al., 2018 (first-in-human hybrid IVUS-OCT, *Novasight HybridTM* system); Jia et al., 2023 (the *PANOVISON* catheter); Zhao et al., 2025 (OUFR); Jin et al., 2025 (multicenter study, *Novasight HybridTM* system)	Fard et al., 2013 (Ex vivo validation); Ali et al., 2024 (In vivo validation; HyperVue Imaging System, SpectraWAVE, Inc)	Schultz et al., 2010 (first-in-man clinical use); PROSPECT II, (NIRS-IVUS identify high-risk plaques and patients that are at risk for MACEs)	Wang et al., 2012 (IVPA/IVUS imaging of lipid distribution); Kole et al., 2018 (first comparison of IVPA-IVUS and NIRS-IVUS for arterial lipid quantification); Lin et al., 2021 (targeted IVPA/IVUS imaging of angiogenesis in progressed atherosclerotic plaques);	Yoo et al., 2011 (in vivo validation); Verjans et al., 2016 (clinical and intracoronary evaluation of indocyanine green); Kim et al., 2024 (OFDI-NIRF imaging-assisted intravascular theranostic photoactivation of atherosclerotic plaque)	Lee et al., 2018 (imaging of atherosclerotic plaque in rabbit artery); Kim et al., 2025 (first-in-Human OCT-FLIm)	Fatakdawala et al., 2015 (FLIm-IVUS improved plaque characterization accuracy, including TCFA detection); Bec et al., 2017 (FLIm-IVUS can assess collagen and elastin and their degradation)	Leng et al., 2021 (first developed)
**Clinical Applications**	Luminal and vessel wall pathology imaging; PCI optimization	Culprit plaque characterization; PCI guidance; ACS assessment	Lipid burden quantification; mild non-flow-limiting lesion identification; PCI guidance	Detection of plaque lipid composition, neovascularization, and inflammation (based on the characteristic optical absorption)	Image and quantify plaque inflammation (augmented NIRF cysteine protease activity); simultaneous mapping of plaque structure	Multitargeted characterization of high-risk plaque (the lifetime of fluorescence emissions)	Simultaneous assessment of plaque morphological and biochemical features	**/**
**Key Advantages**	More accurate detection of high-risk plaques; versatility for complex bifurcations	Enable the correlation between lipid content and OCT microstructural features; automated lipid lesion segmentation	Quantitative lipid assessment (maxLCBI_4mm_)	Depth-resolved characterization of atherosclerotic plaques	Combine microscopic features of OCT and molecular information including inflammatory protease activity, macrophage composition; adhesion molecules, and fibrin	Combine microscopic features of OCT and molecular FLIm features including macrophages, healed plaques, calcification, and fibrosis	FLIm feature dimensions (similar to OCT-FLIm) can be precisely assessed when projected onto the IVUS-defined lumen boundary	Identify and quantify lipids using multi-wavelength excitation
**Current Limitations & Challenges**	Miniaturization; co-registration; limited image acquisition speed high cost	Insufficient in vivo validation	IVUS: limited spatial resolution and shadowing artifacts; NIRS: absence of lipid core depth information	Catheter size and flexibility limitations; unsuitable for real-time clinical imaging	Limited penetration depth of OFDI and NIRF; only semiquantitative measurement of plaque molecular concentration	The requirement to clear blood; low penetration depth	The requirement to clear blood; low penetration depth due to the use of UV light; limited image acquisition speed	Limited imaging speed; motion artifacts; catheter rigidity
**Ref**.	^103,108,221,222,304–308^	^106–108,222,309,311^	^57,109–111,310,312^	^112,222,313–315^	^111,113,114,316^	^111,115,280^	^116,317,318^	^117^

*OUFR* optical ultrasonic flow ratio, *maxLCBI*_*4mm*_ maximum lipid core burden index within a 4-mm coronary section, *MACE* major adverse cardiovascular events, *OFDI* optical frequency domain imaging, *NIRF* near-infrared fluorescence, *FLIm* fluorescence lifetime imaging

#### IVUS-OCT

**Fig. 6:**
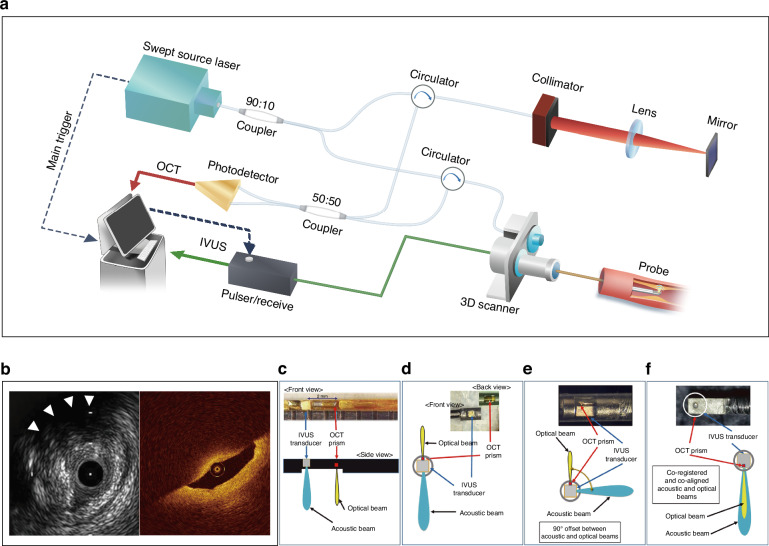
Schematic of the integrated dual-modality OCT-IVUS system. **a** Overview of a typical OCT-IVUS system architecture. **b** Representative co-registered IVUS (left) and OCT (right) images. (reprinted with permission from ref. ^304^ ©2015 Elsevier) **c**–**f** Schematics of distinct probe configurations. (reprinted with permission from ref. ^103^ ©2021 Elsevier) **c** Sequential arrangement; **d** Back-to-back arrangement; **e** Co-planar arrangement with a 90° offset; and **f** Collinear arrangement

Hybrid OCT-IVUS imaging integrates OCT assessment of fine surface details with IVUS visualization of deeper structures, enabling a more precise characterization of the type of plaque and the identification of TCFAs (Fig. 6b).

Figure 6a illustrates the overall architecture of a typical IVUS-OCT fusion imaging system. The drive module serves as the interface between the catheter and the imaging system, housing a rotary motor, a linear pullback stage, and signal coupling connectors. The imaging console comprises multiple hardware and customized software to control the transmission, reception, and processing of both IVUS and OCT signals^103^.

Probe design has evolved significantly to optimize performance^103–105^: (1) Early designs (Fig. 6c): A sequential arrangement was adopted. However, the longitudinal offset between the ultrasonic and optical imaging cross-sections resulted in suboptimal image registration. (2) Co-planar design (Fig. 6e): Subsequently, researchers integrated the OCT prism and ultrasound transducer side-by-side within a 4 Fr probe. Here, the acoustic and optical beams scan the same tissue cross-section with a 90° offset, allowing for more precise simultaneous registration even during cardiac motion. (3) Back-to-back configuration (Fig. 6d): This design further improved frame rates and facilitated catheter miniaturization. (4) Collinear design (Novasight Hybrid™, Fig. 6f): Recent systems utilize a collinear approach, where acoustic and optical beams are aligned. This allows for simultaneous imaging of the exact same cross-section, effectively eliminating registration artifacts caused by cardiac motion or variations in rotational speed.

#### OCT-NIRS

The synergistic advantage of combined OCT-NIRS imaging lies in complementarity: spectroscopy detects the presence of lipids, while OCT characterizes structural features, specifically fibrous cap thickness, thereby localizing the depth of the lipid core identified by NIRS.

**Fig. 7:**
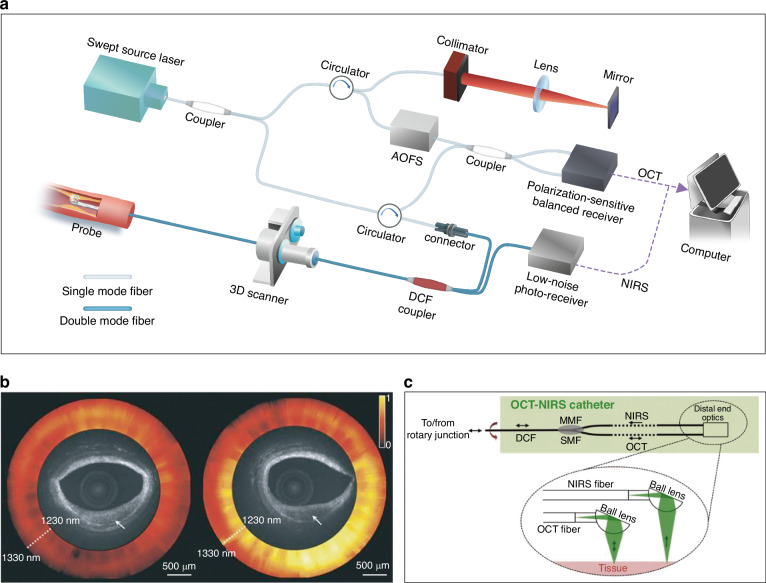
Integrated dual-modality OCT-NIRS system and imaging. **a** Schematic illustration of a typical OCT-NIRS system architecture. AOFS: Acousto-optic frequency shifter. **b** Representative ex vivo OCT-NIRS images derived from a human coronary artery. **c** Schematic design of the hybrid OCT-NIRS catheter. (reprinted with permission from ref. ^106^ ©2013 Optica Publishing Group)

Figure 7a schematically shows a typical integrated dual-modality OCT-NIRS system. A representative catheter-based OCT-NIRS hybrid system employs a dual-fiber architecture in conjunction with a wavelength-swept light source to enable synchronous acquisition of structural and spectroscopic information. OCT signals are delivered and detected through the fiber core, whereas NIRS signals are collected via the inner cladding; the two channels are subsequently demultiplexed by a fiber-optic coupler and routed to their respective detectors. Simultaneous acquisition of OCT and NIRS signals makes it possible to obtain one spectrum per axial scan, facilitating spatial registration of the two data sets^106^.

At the catheter’s distal tip, two angle-polished ball lenses are configured so that the core provides OCT imaging, while the cladding samples diffuse light approximately 1–2 mm from the illumination point, interrogating absorption spectra at depths of 500–1500 μm (Fig. 7c). This optical architecture ensures that OCT morphological data and NIRS compositional information are synchronous and spatially co-registered within each A-line, establishing a robust platform for dual-modality plaque assessment^106,107^ (Fig. 7b).

#### IVUS-NIRS

Combined NIRS-IVUS imaging is the only hybrid IVI technology currently approved for clinical use^108^. Clinical evidence indicates that by assessing features such as plaque cavities, convex calcium, and max LCBI, NIRS-IVUS can accurately distinguish between plaque rupture, plaque erosion, and calcified nodules, thereby aiding in the prediction of adverse events^109^.

**Fig. 8:**
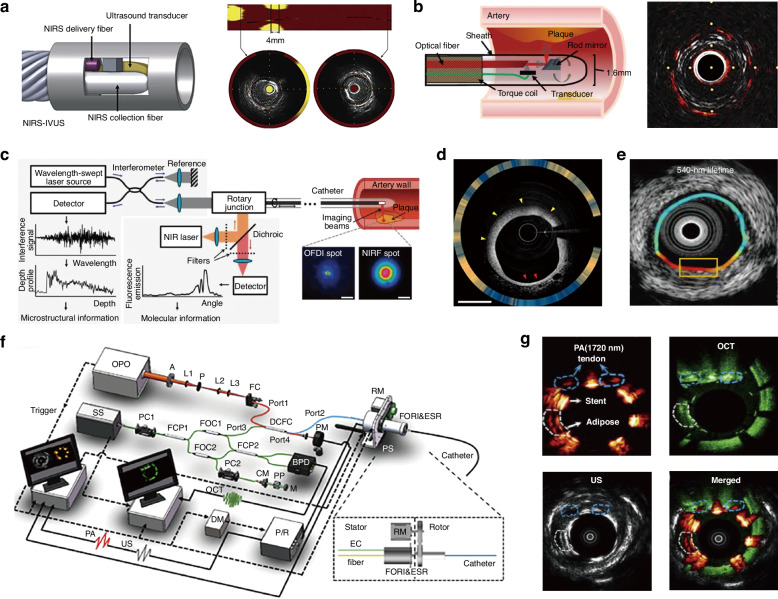
Schematics and imaging results of integrated multimodality systems. **a** Schematic of the IVUS-NIRS catheter tip assembly (left) alongside a NIRS chemogram and co-registered cross-sectional view (right) (reprinted with permission from ref. ^111^ ©2022 Elsevier). **b** Collinear IVPA-US catheter design (left) and composite imagery showing depth-resolved lipid signals localized to the perivascular region (reprinted with permission from ref. ^112^ ©2019 Springer Nature). **c** Schematic of the dual-modality OFDI-NIRF catheter designed for simultaneous microstructural and molecular intravascular imaging (reprinted with permission from ref. ^113^ ©2011 Springer Nature). **d** In vivo FLIM-OCT imaging of atherosclerotic plaque in a rabbit aorta (reprinted with permission from ref. ^280^ ©2018 Springer Nature). **e** Fluorescence intensity (540 nm) combined with IVUS (FLIM-IVUS) characterizing macrophage foam cell infiltration (reprinted with permission from ref. ^116^ ©2021 Elsevier). **f** System overview of the novel Multi-Spectral Intravascular Tri-Modality (MS-IVTM) platform (reprinted with permission from ref. ^117^ ©2021 Optica Publishing Group). **g** Representative photoacoustic, OCT, and IVUS images, along with the fused tri-modality image (reprinted with permission from ref. ^117^ ©2021 Optica Publishing Group

The NIRS-IVUS system has evolved into a dual-frequency, dual-modality platform. The ultrasound transducer and NIR fiber array are integrated within a single catheter, typically utilizing a coaxial architecture, to facilitate synchronous signal acquisition and precise spatial co-registration. The catheter tracks over a standard 0.014-inch guidewire and features a 50-cm hydrophilic-coated distal segment to negotiate tortuous lesions. During automated pullback, the system simultaneously acquires IVUS imagery and spatially co-registered NIRS measurements (Fig. 8a). The resulting lipid data is mapped onto a chemogram, from which LCBI is derived to quantify the distribution and burden of lipid-rich plaques within the target vessel^110,111^.

##### Emerging dual- and tri-modality technologies

In parallel, other hybrid imaging technologies are expanding the diagnostic landscape:

**IVPA-US (**Fig. 8b**):** Intravascular Photoacoustic-Ultrasound imaging offers lipid sensitivity comparable to NIRS-IVUS but with superior depth resolution, enabling precise quantification and localization of the lipid core^112^.

OFDI-NIRF: Near-infrared fluorescence (NIRF) imaging exploits photoluminescence to identify molecular signatures associated with plaque inflammation. To date, indocyanine green (ICG) is the only FDA-approved NIRF agent^111^. Figure 8c illustrates an intra-arterial dual-modality catheter designed for simultaneous microstructural and molecular imaging in vivo^113^. In vivo validation has demonstrated that this system effectively detects ICG deposition within macrophage-rich atherosclerotic plaques^113^, and recent work has further substantiated its potential for catheter-based theranostic applications^114^.

**FLIm-IVOCT (**Fig. 8d**), and FLIm-IVUS (**Fig. 8e**):** FLIm uses UV light to excite fluorophores and can distinguish multiple biochemicals of interest. FLIm OCT has already had human use, while FLIm-IVUS has undergone preclinical studies. Kim et al. utilized hybrid FLIm-IVOCT system in first-in-human studies to characterize plaque microstructure and molecular signatures, including macrophages, healed plaques, calcification, and fibrosis^115^. Bec et al. employed FLIm-IVUS system to quantify and predict superficial macrophage foam cell infiltration in human coronary segments^116^.

Furthermore, tri-modality systems represent the next frontier in comprehensive plaque assessment:

**MS-IVTM (**Fig. 8f, g**):** Developed by Ji Leng’s team, the Multi-Spectral Intravascular Tri-Modality (MS-IVTM) catheter integrates photoacoustic, ultrasound, and OCT technologies. While currently in the laboratory phase, it shows promise for the holistic assessment of necrotic cores, fibrous cap integrity, and lipid deposition^117^.

Although multimodal fusion technology significantly reduces surgical complexity and improves the accuracy of imaging analysis, its development still needs to overcome a series of challenges: (1) Device and technical design and compatibility: Integrating components with different mechanical structures and pullback speeds requires redesigning the catheter structure. (2) Catheter size: Existing catheters already face size issues, and fusion catheters, due to their more complex mechanical structure, are often no smaller than single-modality catheters, which may hinder their passage through narrow or distal arteries. (3) Acoustic and optical interference: Modality fusion must overcome interference from different acoustic and optical signals and light source wavelengths. For example, electromagnetic interference in an OCT-IVUS catheter can lead to increased noise (speckle) in the IVUS image^111^. (4) Modeling accuracy: After obtaining multi-dimensional imaging results from a multimodal catheter, the inner wall of the vessel must be reconstructed synchronously to ensure that data from different modalities are not spatially misaligned, requiring the use of AI for data reconstruction and analysis.

Critically, even when these engineering challenges are resolved, multimodal fusion does not automatically translate into a unified Suitcordance evaluation across vascular beds. Coronary, peripheral and cerebral arteries differ markedly in caliber, hemodynamics, plaque composition and motion artifact, so a fusion catheter optimized for short-term coronary suitability may not provide a comparable readout of long-term concordance in tortuous peripheral or small-caliber cerebral vessels. Achieving truly bed-agnostic Suitcordance therefore requires not only modality integration but also bed-specific calibration of imaging biomarkers and decision thresholds, which remains an open problem.

## Intra-procedural guidance and AI support

The application of IVI across different vascular beds faces fundamental physical challenges, including cardiac motion artifacts^118–121^, the penetration–resolution trade-off and motion-related artifacts^122^. These physical bottlenecks remain unresolved under conventional paradigms. Computational imaging offers a transformative solution by shifting IVI from passive “visual data acquisition” to active “intelligent information reconstruction”, enabling precise extraction of clinically relevant features from raw optical signals. AI plays a uniquely powerful role: it can address the exponential growth in imaging data throughput that has outstripped conventional computing power, suppress unwanted interference (e.g., blood scattering noise), and integrate information from multiple modalities, and distill imaging patterns to assist image reading and decision‑making. Three core capabilities are provided during procedures: accelerating high-fidelity image reconstruction to overcome hardware limitations, enabling intelligent analysis of imaging results such as automated lesion detection and characterization, and extracting latent patterns to deliver decision support that assists operators in real-time strategy formulation. The following subsections elaborate on these three facets of AI empowerment, followed by a discussion on interventional surgical robots.

### High-fidelity computational imaging and reconstruction

With improvements in the clarity and information content of IVI and the development of multimodal fusion, IVI technologies in the future will increasingly require AI computing power to achieve low-latency, low-noise, high-definition image reconstruction and processing, including accelerated raw signal reconstruction, 3D vessel reconstruction, stent strut segmentation and modeling, and contrast-agent-free imaging.

#### AI for raw signal reconstruction

The feasibility of deep learning for OCT image reconstruction has been demonstrated in several studies, which helps improve resolution and reconstruction speed. Li et al.^123^ proposed a spectral-spatial based sampling method to boost efficiency of image acquisition, while maintaining high performance and flexibility in reconstruction. More broadly, AI-assisted OCT reconstruction has been extensively studied across various OCT modalities, highlighting its strong potential for translation to intravascular OCT^124–130^.

In terms of accelerating image reconstruction, a novel Deep learning-based OCT framework developed by Zhang et al.^126^ effectively accelerated the reconstruction of SD-OCT images without compromising resolution or signal-to-noise ratio, and without requiring any hardware modifications. 3D convolutional neural networks (CNNs) were leveraged by Rashidi et al.^128^ to mitigate information loss and preserve fine details and boundaries in OCTA images. For image denoising, generative adversarial network was employed by Dong et al.^129^ for image denoising, which effectively reduces speckle noise while maintaining spatial and temporal resolutions. Although most current research focuses on OCT, several studies have explored IVUS image reconstruction^131,132^, For example, one approach utilized spatial and temporal information hidden in sequential frames to recover high-fidelity original content and maintain inter-frame consistency^131^.

#### AI for 3D vessel reconstruction

3D vessel reconstruction recovers the 3D anatomy, facilitating quantitative analysis and stenosis localization^133,134^, meanwhile largely improving image readability. Based on a collaborative constraint generative adversarial network, Xia et al.^133^ proposed collaborative constraint generative adversarial network (GAN) named AwCPM-Net, which innovatively links the cardiac phase retrieval and the membrane border extraction, demonstrating reliable results in 3D arterial anatomical reconstruction. Qin et al.^134^ developed a Hybrid-dimensional (HyD) spatial matching network, training with style transfer, which provides accurate 3D segmentations of stents and lumens, and information on stent-to-intimal apposition, largely improve the readability.

#### AI for stent strut segmentation and modeling

**Fig. 9:**
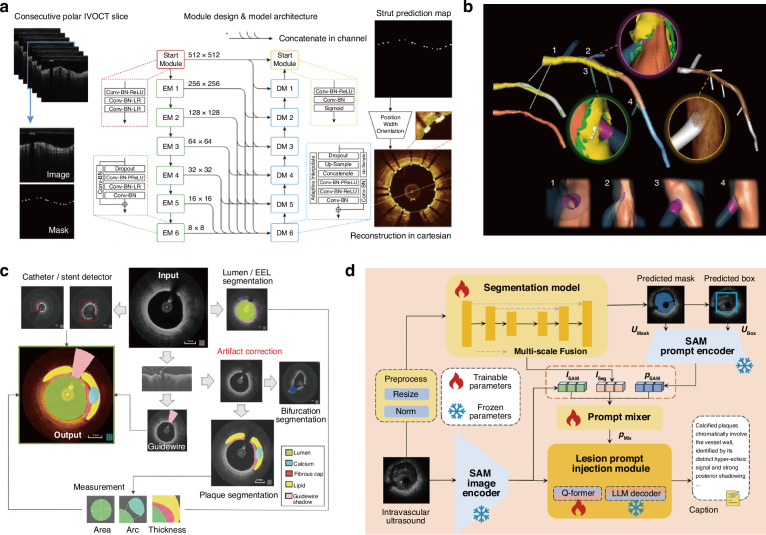
Application examples of AI-assisted image reconstruction and recognition. **a** The input image, overall model architecture and output strut map of the stent strut segmentation model. Pseudo 3D consecutive polar slices to feed the stent strut segmentation model as input, a dedicated model architecture designed for stent strut segmentation, and the strut map predicted from the model (reprinted with permission from ref. ^135^ ©2020 Optica Publishing Group). **b** Example of AutoFOX final fusion result with bifurcation structure, plaques and a 3D-image (reprinted with permission from ref. ^143^ ©2025 Elsevier). **c** Overview of deep learning model of AutoOCT. Input axial OCT frames were preprocessed before undergoing parallel processing. Catheter and stent locations were first classified with frame locations output. Plaque components were then segmented in either polar transform or Cartesian images^157^ (©2025 Jessney et al., licensed under CC BY 4.0). **d** The overall architecture of the PlaqueCap framework, including a segmentation model for lesion localization, frozen SAM encoders for feature and prompt extraction, a prompt mixer, and a Lesion Prompt Injection module for caption generation (reprinted with permission from ref. ^159^ ©2025 Springer Nature)

Beyond the vessel wall itself, deep learning algorithms can also assist in the 3D reconstruction of implanted stents, enabling reliable and real-time analysis during the procedure by segmenting stent struts in IVI frames, assisting in intra-procedural stent evaluation^135,136^. For instance, Wu et al.^135^ proposed a deep convolutional framework leveraging pseudo-3D images and multi-scale shortcut connections for the precise detection of stent struts in IVOCT imagery. In independent testing, the model demonstrated superior segmentation performance (Dice coefficient: 0.907; Jaccard index: 0.838) and robust detection metrics (Precision: 0.943; Recall: 0.940; F1-score: 0.936), significantly outperforming conventional feature-based algorithms. The model architecture is illustrated in Fig. 9a.

#### AI for contrast-agent-free imaging

Furthermore, AI is expected to overcome or partially overcome OCT’s dependence on contrast agents by using deep learning to distinguish between blood flow scattering noise and vessel wall signals, a significant step forward for patients with renal insufficiency.

Beyond purely data-driven architectures, the integration of physics-informed neural networks (PINNs) represents a paradigm shift in addressing the ill-posed inverse problems inherent in panvascular optical imaging. In clinical practice, large-scale annotated datasets are extremely scarce and costly to obtain, rendering purely data-driven models brittle across diverse patients and imaging conditions. Unlike black-box models that rely solely on massive annotated datasets, PINNs constrain the hypothesis space of neural networks by explicitly embedding optical governing equations, such as wave propagation models and scattering physics, directly into the loss functions. This design not only ensures physically consistent reconstructions under noisy or undersampled measurements, but also markedly improves cross-patient and cross-scenario robustness. Consequently, PINNs deliver images that clinicians can trust as actionable diagnostic evidence, directly narrowing the gap between raw sensor output and reliable periprocedural decision-making^137–140^.

AI greatly improves the accuracy, speed, and flexibility of aligning images from different modalities^141^. Previous methods required precise hardware synchronization, which restricted catheter design and made accuracy difficult to adjust^117^. Deep learning, CNNs in particular, is inherently suited to medical image analysis because it automatically learns hierarchical features—from local textures to global anatomy—without manual crafting. This capacity enables fast, robust, and flexible alignment of multimodal intravascular images^142^. Moreover, IVI can also be fused with complementary non-invasive technologies. For example, Fig. 9b shows AutoFOX, an automated 3D fusion framework developed by Li et al.^143^, which employs a deep learning model (TransCAN) to achieve 3D fusion of coronary X-ray angiography and OCT to reconstruct the vessel. This framework enables the simultaneous and accurate assessment of fractional flow reserve (FFR) and endothelial shear stress, holding significant value for guiding interventional procedures and optimizing treatment.

However, model development is highly reliant on high-quality annotated data, and medical data faces issues of annotation scarcity, privacy protection, and lack of standardization, which limits model training and promotion. Additionally, the generalizability of existing algorithms across different devices, populations, and even vascular regions remains insufficiently validated and their robustness has not been fully confirmed clinically. As data sharing improves and AI methods that require less annotation mature, the clinical utility of AI-enhanced IVI will expand significantly. In the future, we can also expect to see further collaboration between AI-photonics and IVI. For instance, integrating analog optical computing or on-chip photonic neural networks directly at the catheter tip could revolutionize data handling. These photonic integrated circuits enable zero-latency image pre-processing and feature extraction at the edge, drastically reducing the bandwidth requirements for back-end transmission and enabling the deployment of lightweight AI models to further reduce back-end computational load and achieve low-latency, high-quality intra-procedural image reconstruction^144^. Looking beyond purely data-driven approaches, PINNs represent a pivotal frontier. When integrated with the analog optical computing at the catheter tip, this physics-aware paradigm can solve inverse imaging problems at the edge in real time, breaking current limits in imaging speed and resolution. This convergence of hardware-level photonics and embedded physical intelligence promises to realize a new generation of catheters with intrinsic optical reasoning capability.

### AI-supported rapid intra-procedural structure segmentation and lesion identification

Traditional analysis of IVI results not only relies heavily on the operator’s experience but is also limited by the operator’s analytical capabilities, causing a large amount of data to be overlooked. AI can overcome the limitations of traditional pathological analysis. It has shown great potential in automated image segmentation, plaque identification, and vascular stenosis assessment, improving the efficiency and accuracy of diagnosis and treatment. This will effectively reduce the dependence of IVI on operator experience and promote the more comprehensive utilization of the burgeoning volume of IVI data, laying the foundation for the wider adoption of IVI and the improvement of diagnostic accuracy and treatment guidance.

#### AI in structure segmentation

Studies have validated the efficacy of deep learning in IVI tissue segmentation, especially in IVUS. This includes the precise delineation of normal anatomical structures, specifically the lumen, media, and adventitia, as well as the identification of dense calcified tissue, the quantification of signal attenuation, and the assessment of intra-plaque calcification burden^145–153^. Architectures like GAN, U-Net and Transformer have been broadly employed^151–153^. GANs can synthesize realistic intravascular images, serving as a data augmentation strategy to alleviate training data scarcity^154^, and also facilitating domain adaptation^152^; U-Net automates precise anatomical and device segmentation through its encoder-decoder framework^155^, and Transformers capture global context via self-attention to handle complex morphologies^151^, thereby achieving high segmentation accuracy, improved observer consistency, and enhanced robustness to shadowing and complex morphology, thereby streamlining intra-procedural workflow for the operator.

#### AI in lesion recognition

Beyond structural segmentation of imaging results, numerous techniques have been developed for fine-grained lesion recognition, providing critical information for diagnosis and treatment, including plaque composition and vulnerability, thereby offering essential decision support to operators^150,154,156–158^. For instance, in addition to correcting segmentation errors in low-quality or artifact-laden OCT images, AutoOCT, a deep learning-based module, enables tissue and plaque composition identification, categorizes plaque phenotypes, and quantitatively assesses critical parameters, including lumen area, lipid and calcium arcs, and fibrous cap thickness, as illustrated in Fig. 9c. This comprehensive dataset supports clinical decision-making, significantly enhancing procedural suitability^157^. PlaqueCap, a lesion-centered captioning framework, integrates a high-fidelity segmentation module with a language generation pipeline, producing detailed, clinically interpretable descriptions of lesion location and classification, which surpasses baselines in quantitative metrics and expert evaluation^159^. Its architecture is shown in Fig. 9d. Benefiting from the exceptional image processing capabilities of CNNs, this network can be effectively applied to lesion recognition^160^, for example, predicting neointimal coverage grade, plaque yellow color grade, and the presence of red thrombus in Angioscopy images, which significantly reduces the adverse impact of operator subjectivity on lesion identification^154^. Amid the exponential expansion of data generated by modern IVI technologies, AI is uniquely positioned to comprehensively harness this information to derive robust diagnostic conclusions, effectively mitigating the risks of omission and misinterpretation arising from operator subjectivity.

The development of multimodal fusion technology presents new challenges for operators’ image interpretation skills, and AI-assisted pathological identification is expected to mitigate this negative effect. As technologies like Contrastive Learning, GNN, and Transformer are flexibly applied to intra-procedural lesion identification, the reliance of IVI on operator experience will continue to diminish, data utilization will be more comprehensive, and operators will be able to formulate more informed and accurate treatment plans based on the identification results.

In addition to lesion identification, the introduction of AI allows for the evaluation of metrics such as image segmentation and model stability using coefficients like the Dice Coefficient, AUC (area under the curve), and F1-Score. This provides clinicians with reliable quantitative evidence, which is essential for building a trustworthy clinical support system^161–164^.

It should be noted, however, that high segmentation accuracy and high lesion-recognition AUC do not by themselves equate to a valid Suitcordance assessment. A Dice or F1 score reports geometric agreement with a reference annotation, whereas Suitcordance requires translating those measurements into device–vessel matching decisions (stent sizing, landing zone, post-dilation strategy) and into predictions of long-term vascular response. The residual gap between pixel-level technical performance and clinical decision support, particularly for safety-critical features such as edge dissection or thrombus-versus-artifact discrimination, therefore remains a first-order obstacle to AI-driven Suitcordance, and is a recurring theme in the remainder of this section and in the outlook.

### AI-assisted intra-procedural treatment decisions

Despite advances in interventional techniques and stent technology, acute stent thrombosis, in-stent restenosis, and stent underexpansion remain persistent challenges compromising procedural success. By integrating AI-assisted image recognition functions, AI empowerment can provide guidance for interventional procedures, improve surgical precision, and play an important role in delicate and complex surgical environments like the coronary, carotid, and intracranial arteries. It can assist operators in making informed surgical decisions based on the vascular environment, such as prediction of stent expansion outcomes and other treatment result, thereby emerging as a vital instrument for clinical decision support^165^.

#### AI in stent deployment planning

For instance, Wu et al.^166^ developed a deep learning architecture termed the “Dual-Branch Medical Image Detector” (DBMedDet). Aimed at facilitating real-time intraoperative guidance, this model achieved a mean average precision (mAP) of 0.841 (at mAP@0.5) with a throughput of 127 frames per second (FPS). Its versatility across diverse vascular territories, including coronary, peripheral, and cerebrovascular beds, offers enhanced procedural precision and safety, ultimately leading to improved therapeutic outcomes and quality of life for patients. Colmenarez et al.^167^ developed a diffusion model, which gives operators real-time, FEM-equivalent predictions of stent expansion and rupture risk from IVOCT, enabling on-the-spot decisions on balloon sizing, plaque modification, and stent landing without complex simulations. Deep learning algorithms based on IVUS can reliably predict the degree of expansion after stent deployment, which can help clinicians make treatment decisions and reduce the risk of procedural failure due to incomplete stent expansion^168^.

#### AI in lesion risk prediction

Lesion risk prediction is based on precise lesion recognition. Intra-procedural lesion risk prediction helps guide interventional decision-making and reduce potential adverse events. Lv et al.^169^ introduced quantitative plaque cap thickness and stress/strain indices to quantify plaque vulnerability, and employed a machine learning method (random forest) to assess and predict vulnerable plaque progression and rupture risk, achieving high prediction accuracy with multimodal IVUS and OCT image. The models mentioned above are compared in detail in Table 4.

**Table 4:** AI models for intra-procedural decision support

	DBMedDet by Wu et al.^166^	Plaque progress prediction model by Lv et al.^169^	Denoising diffusion model for post-PCI prediction by Colmenarez et al.^167^	Stent deployment prediction model by Min et al.^168^
**Targeted Clinical Problem**	Challenges in stent deployment planning	Difficulty in plaque risk prediction	Difficulty to predict post-PCI atherosclerotic artery biomechanics	Difficulty to predict stent underexpansion and stent failure
**Model Architecture**	A parallel dual-branch edge feature extraction network, integrating YOLOv8, Sobel operator, RMNet	Random Forest, 50 trees, 5-fold cross-validation, implemented via MATLAB TreeBagger	Denoising diffusion probabilistic models (DDPM) and conditional image generation	CNN to predict stent area, binary classification models (XGBoost) for sections classification
**Input Modality**	Intraoperative DSA image	Multimodality IVUS + OCT data	Pre-stenting IVOCT images	IVUS frames
**Clinical Decision Assistance**	Real-time constraint cues for the optimal placement of stents	Quantitative plaque vulnerability indices (cap-thickness, stress, strain based)	Enabling full 3D reconstruction of post-PCI morphologies, showcasing lumen expansion, assessing the risk of calcification and fibrous cap rupture	Predicted incomplete stent expansion, assist clinicians’ treatment decisions to avoid underexpansion
**Key Quantitative Results**	mAP@0.5: 0.841; real-time detection speed: 127 FPS; superior performance compared to several YOLO	Cap-PVI: 90.3% (AUC = 0.877), mean cap stress PVI: 85.6% (AUC = 0.867), mean cap strain PVI: 83.3% (AUC = 0.809)	Structural similarity index >0.894, learned perceptual image patch similarity <0.048, deviations of 6.86% from FEM area profile curves (lumen expansion accuracy)	Maximal accuracy of 94% with CNN + mask image-derived features; predicted vs. measured: min stent area, *r* = 0.832, total stent volume, *r* = 0.958

*mAP@0.5* mean average precision when IoU threshold = 0.5, *PVI* plaque vulnerability indices, *FEM* the finite element method, *CNN* Convolutional Neural Networks

The high-resolution, real-time, three-dimensional vessel-wall information delivered by IVI provides the indispensable data foundation for constructing an intra-procedural “digital twin” of the vascular environment. A medical digital twin is a computational model that mimics the behavior of an organ system while retaining predictive capability, and it is increasingly positioned to deliver critical intra-procedural decision support. Extensive work has been reported across multiple medical fields^170–176^, and early exploration has begun in the panvascular domain^174,175^.

IVI can precisely delineate plaque composition, calcium distribution, vascular remodeling status and the microscale stent-vessel-wall interaction; once parsed in real time by AI models, this rich histological and geometric information can be injected into patient-specific multi-physics simulations that dynamically predict how alternative interventional strategies, stent sizing, landing-zone planning, post-dilation pressure, will affect acute lumen gain, strut apposition and local haemodynamics. This “sensing-simulation-feedback” closed loop allows the operator to rehearse and optimize decisions in a virtual space, elevating intervention from experience-driven practice to model-driven precision.

Nevertheless, realizing digital-twin technology places stringent demands on data quality, scale and model generalizability. Laboratory datasets are constrained by acquisition conditions and sample distribution and therefore often generalize poorly to the clinical setting, while clinical datasets commonly suffer from incomplete information, heterogeneous quality and limited volume. IVI-AI models trained and validated on high-quality, standardized coronary pullback data, for example, exhibit substantial performance degradation when confronted with the complexity of real-world peripheral or cerebral interventions, and therefore struggle to supply reliable inputs to the digital twin. Several strategies can improve data generalizability, including data-augmentation techniques^177^, domain-adaptation training^152,178^ and the PINNs to impose prior physical constraints^172^, thereby strengthening model robustness against unseen data distributions. Establishing ethics-approved, rigorously de-identified, standardized multi-center shared databases would further enrich the diversity and representativeness of training samples at the source, alleviating the generalization bottleneck introduced by single-center bias. In addition, the clinical translation of AI still faces systemic obstacles, including the opacity of the “black box”, a lengthy and complex regulatory-approval pathway, high costs for continuous algorithm maintenance, uncertain real-world cost-effectiveness, and the difficulty of integrating AI stably and seamlessly into existing clinical workflows. Collectively, these challenges constrain the journey of AI-enabled digital twins from proof-of-concept to clinical reality.

### Development of interventional surgical robots

Interventional surgical robots can not only reduce occupational hazards in this field, such as radiation exposure and orthopedic risks, but also significantly improve operational precision, reduce operator subjective errors, and lower the risk of intra-procedural complications^179^. They can also enable remote intervention, thereby expanding access to specialized care and reducing the need for on-site physician presence during emergencies. Systems like the CorPath 200 have been successfully used in robot-assisted PCI (R-PCI) for complex coronary artery lesions, and were approved by the FDA for PCI in 2012^180^. Related multi-center studies have shown a technical success rate of up to 98.8% and a clinical success rate of about 97.6%, while significantly reducing the operator’s radiation exposure^181^.

Although robots for cardiovascular intervention were developed relatively early, it was not until 2019 that the CorPath GRX began to be applied in neurointerventional clinics^182^, due to the more fragile nature, smaller caliber, and greater structural complexity of cerebral vessels. Its development must also be closely integrated with AI and optical technologies, achieving universal applicability in the panvascular environment through synergistic development with AI-powered path analysis, optical sensing, and other techniques. AI technology can analyze massive amounts of medical data and provide real-time feedback to the robot, thereby offering more accurate predictions and surgical plans, gradually closing the perception-decision-execution loop and demonstrating significant advantages in clinical interventional therapy.

#### AI and optics for perception enhancement

Innovations in IVI and their synergistic integration with AI are expected to contribute to the visual guidance of interventional robots. Multimodal fusion and optical catheter innovations can achieve more comprehensive and clearer imaging of the vessel wall, providing more robust data support for pathological analysis and treatment decisions.

Currently, interventional surgical robot systems are primarily guided by vision, using multiple intra-procedural imaging modalities to provide visual guidance for guidewire advancement and lesion localization. To address the lack of force perception in interventional robots, miniaturized and high-precision fiber-optic mechanical sensing technologies can monitor the interaction force between the device tip and the vessel wall in real-time via optical wavelength shift mechanisms, thereby significantly enhancing operational safety and precision^183^. In recent years, robotically steerable guidewires have advanced traditional guidewire guidance, which relies on tactile feedback from wall contact and external imaging, to a new paradigm that is visualizable, remotely controllable, and capable of full force sensing^184–186^. With the development of AI and various optical imaging modalities, it is anticipated that in the future, the deep integration of AI and optics will enable real-time intra-procedural catheter tracking, more comprehensive imaging, and a closed loop of perception and control framework.

#### AI in perceptual decision loop

The integration of AI can provide comprehensive assistance in image analysis, lesion identification, decision support, and strategy optimization, gradually moving toward intelligent closed-loop control. In terms of decision-making for interventional robots, the application of AI is still at an early stage^187^. The analytical capabilities for large datasets and the rapid lesion identification provided by AI can offer important references for clinicians’ surgical planning. However, due to difficulties in data acquisition, the need to rigorously validate reliability, and unresolved liability concerns, AI is still far from being able to execute autonomously decisions and will require sustained, collaborative development across various fields.

At the execution level, with the development of panvascular diagnosis and treatment, interventional surgery demands even higher levels of precision, such as for treating complex lesions in the distal cerebral cortex. Although surgical robots possess excellent procedural accuracy, they still require physician supervision and guidance. Some research on magnetically controlled interventional robots may offer promising breakthroughs in fine-grained control. Wang et al.^188^ designed a stent-like, soft, untethered surgical robot device that is small enough to adapt to vascular morphology and reach distal vessels inaccessible to traditional interventions, offering a novel minimally invasive and targeted treatment method for distal and tortuous vessels, including for intracranial aneurysms and arteriovenous malformations. AI assistance and some cutting-edge optic technologies, such as optical phased arrays, LiDAR and near-field optical sensors, hold promise for application in interventional surgical navigation, enabling non-contact 3D spatial modeling and minimally invasive path guidance^189–191^.

Concurrently with achieving information acquisition and analysis with optics, future interventional robots are expected to incorporate additional optical therapeutic modules at the execution end. For instance, upon identifying an atherosclerotic plaque, they could utilize integrated photodynamic or photothermal therapy modules to stabilize the lesion in situ, thereby improving surgical success rates and outcomes. Although these solutions are relatively mature in fields like autonomous driving and phototherapy, their applicability in the highly complex intravascular environment remains unverified and is still at an exploratory research stage.

The development of interventional surgical robots also faces a series of critical challenges that need to be overcome. For example, the tactile feedback of surgical robots is not yet sufficiently sensitive and is limited to the catheter tip^192^, which is particularly disadvantageous in highly delicate and tortuous intracranial procedures; current surgical robots are not fully integrated with existing interventional suite equipment^193^, and surgical robots have limited emergency response capabilities. There are also ongoing medical ethics disputes. As an integration of technologies for catheter devices, real-time analysis and decision-making, and tactile feedback, interventional surgical robots require synergistic development in all these areas. In the future, interventional surgical robot systems are expected to integrate multimodal sensing capabilities, including visual perception, optical imaging, magnetic actuation, tactile feedback, and electromagnetic positioning, and combine them with edge AI computing platforms to improve processing efficiency. This will enable supervised and progressively autonomous operations, building a truly integrated “intelligent interventional surgical ecosystem” and providing safer, more efficient, and more precise interventional solutions for vascular diseases.

## Post-procedural assessment and follow-up management

In recent years, intravascular optical imaging technology has evolved from an ancillary imaging tool into a key supportive modality for the precise post-procedural management of PVDs^194,195^. According to our proposed Suitcordance framework, post-procedural management requires balancing short-term suitability and long-term concordance. To systematically organize this concept, we summarize the hierarchical framework in Table 5. This coordinated device-vessel alignment supports the restoration and maintenance of key vascular ecological balances, thereby promoting a microenvironment favorable to long-term vascular health. Leveraging high-resolution structural and functional feedback, intravascular optical imaging develops interventional assessment from static validation to dynamic monitoring, thus closing the loop between short-term efficacy evaluation and long-term follow-up.

**Table 5:** Post-procedural & follow-up summary

	Immediate optimization	Long-term healing & prognostic management	Adjunct/complementary remote monitoring
**Core Objective**	Achieve acute device-vessel geometric suitability and hemodynamic restoration	Ensure vascular healing and biological integration while restoring and maintaining vascular ecological balance	Enable continuous, non-invasive, real-time vascular surveillance
**Targets & Metrics**	Stent expansion: MSA > 5.5 mm^2^ (IVUS) and >4.5 mm^2^ (OCT); malapposition (axial distance <0.4 mm and <1 mm length); no deformation; ED (<60°, flap limited to intima, <2 mm length); plaque burden <50% at stent edge and no lipid pool; no thrombus and protrusion; no GM and inflow/outflow disease	Neointimal coverage (≥90%)& uniformity; neoatherosclerosis; LAM; scaffold discontinuity; non-culprit lesion progression; TCFA features	Arterial stiffness; oxygen saturation (SpO₂); HRV; blood flow perfusion; tissue oxygenation/metabolic signals
**Imaging/Sensing Modalities**	OCT, HF-OCT, IVUS, NIRS; NIRS-IVUS, OCT-NIRF, FLIm-OCT/IVUS;	Serial OCT/IVUS; IVUS-NIRS, OCT-NIRF; PS-OFDI, 4D-OCT flow, PAI	PPG, wearable fNIRS, photoacoustic sensor, laser doppler device
**Management Implications**	Post-dilatation, lesion modification, additional stenting, focal stent optimization, intensified antithrombotic therapy, functional reassessment (FFR/iFR), repeat imaging follow-up, escalated surveillance	Risk-adapted DAPT, post-dilatation intensive lipid-lowering and plaque-stabilizing therapy, risk-based imaging follow-up	Early warning, therapy titration based on PWV, HRV, and SpO₂ trends, telemedicine integration
**Ref**.	^7,11,12,46,319–321^	^239,242,243,322–324^	^253,257–261^

*MSA* minimum stent area, *FFR* fractional flow reserve, *iFR* instantaneous wave-free ratio, *LAM* late-acquired malapposition, *HRV* heart rate variability, *PPG* photoplethysmography

### Short-term suitability

**Fig. 10:**
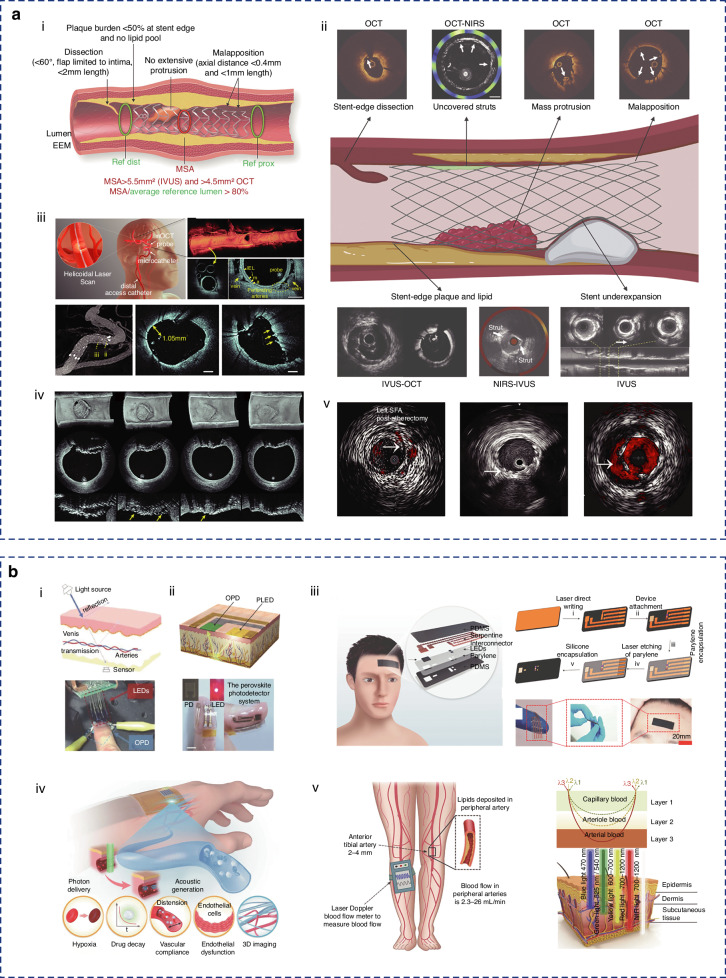
Postoperative intravascular imaging and external monitoring systems. **a** Intravascular/catheter-based follow-ups. (i) Post-PCI optimization targets (reprinted with permission from ref. ^196^, ©2018 Oxford University Press). (ii) IVI for post-PCI coronary monitoring (ref. ^222^, ©2024 Elsevier). (iii) nOCT for neuro-intravascular imaging (ref. ^204^, ©2024 AAAS). (**iv**) HF-OCT imaging of in vivo aneurysm-neck remodeling at baseline vs. 7, 30, and 180 days post-implantation (ref. ^214^, ©2019 BMJ Publishing Group). (**v**) IVUS-guided detection of lower-extremity arterial dissection and stent malapposition (ref. ^205^, ©2022 CongrHealth.com). **b** External/wearable monitoring. (i) Transmissive PPG sensors (refs. ^254^, ©2020 Wiley;^255^, ©2022 Wiley). (ii) Reflective PPG sensors (refs. ^256^, ©2023 Wiley;^253^, ©2025 Springer Nature). (iii) Flexible wearable system for intracranial-hemoglobin monitoring (ref. ^260^, ©2022 MDPI). (iv) Flexible photoacoustic “blood stethoscope” (OBS) for non-invasive cardiovascular monitoring (ref. ^257^, ©2023 Springer Nature). (v) Wearable laser-Doppler flowmeter for peripheral arterial assessment (ref. ^261^, ©2022 Awan et al., CC BY 4.0)

Figure 10a(i) demonstrates the key optimization targets for immediate post-procedural assessment.

Regarding stent under expansion, it is considered one of the primary predictors of in-stent thrombosis and restenosis^31,196,197^. Among IVI parameters, the final minimal stent area (MSA), which reflects absolute stent expansion, has consistently been the strongest predictor of stent patency^198,199^. Both OCT and IVUS can precisely quantify the MSA to assess expansion adequacy and guide further management. For non-left main coronary stents, multiple studies indicate that an MSA < 4.5 mm² (OCT) or <5.5 mm² (IVUS), or a relative stent expansion (i.e., the ratio of MSA vs. the average reference lumen area) <80% (or <90% when using the distal reference as the denominator), is considered a key morphological threshold for increased risk of post-stent thrombosis and restenosis^196,200–203^. Due to the small caliber, tortuosity, and complex hemodynamic environment of cerebral vessels, conventional IVI catheters are limited in post-stent assessment. These challenges have propelled the development of novel designs, such as neuro OCT (nOCT), which combine extremely high resolution with flexible miniaturization to evaluate stent expansion^204^ (Fig. 10a(iii)). In peripheral vessels, such as the femoropopliteal artery with long, multi-segment calcified lesions, IVUS is widely used as the preferred tool for assessing MSA and “apposition gaps” due to its 70 mm field of view, absence of flushing required, and high sensitivity to calcification and metallic echoes^205–207^ (Fig. 10a(v)).

Stent apposition is a critical parameter determining early hemodynamic balance. Computational fluid dynamics and in-vitro models have shown that malapposed regions generate abnormal shear patterns and flow stagnation near protruding struts or in the gap between struts and the vessel wall, which are drivers for thrombus formation, neointimal hyperplasia, and restenosis^208,209^. In cerebral vessels, stent apposition affects peri-procedural stroke risk and the long-term occlusion rates of unruptured aneurysms treated with flow-diverting devices^210,211^. The feasibility of OCT for identifying stent malapposition in clinical practice has been validated^210,212^. Jung et al.^213^ proposed a cerebral-specific OCT (bOCT) catheter, an improved version of the cardiovascular OCT catheter. Through significant structural modifications, its pushability in tortuous vascular paths was markedly enhanced, thereby improving access to cerebral vessels. Concurrently, HF-OCT and nOCT, as high-resolution optical intravascular technologies, can precisely detect stent-to-vessel wall apposition, providing an imaging basis for early post-procedural intervention and anti-thrombotic management^204,214^. For coronary arteries, OCT demonstrates a superior detection rate compared to IVUS (8.0% vs. 0.8%; *P* < 0.001)^215^. In proximal bifurcations or highly curved segments, it can identify sub-millimeter gaps and distinguish between acceptable and correctable malapposition^31,215^. In peripheral segments, IVUS and OCT can display complementary imaging features and assess malapposition more accurately than DSA alone^207^.

The identification of stent edge dissection (SED) and geographic miss (GM) is equally crucial. During stenting, the junction between the rigid stent struts and the compliant vessel wall is susceptible to unintended local tears or dissections, which are associated with post-procedural thrombosis and increased risk of MACE^216,217^. Furthermore, if the lesion or injured segment is not fully covered by the stent (GM)^218^, the residual plaque burden, high-plaque-burden edges, or uncovered segments may serve as nidi for dissection or restenosis^217,219^. Post-procedural identification of SED and GM in coronary arteries relies mainly on IVI, primarily OCT and IVUS. OCT’s high resolution allows for the precise detection of superficial tear length, arc, and flaps, as well as accompanying tissue prolapse or micro-hematomas and uncovered residual plaques. Its detection rate for non-flow-limiting micro-injuries is superior to angiography and IVUS^216,220^. IVUS, with its greater penetration, can reveal calcification, deep hematomas, and tears extending to the adventitia, making it particularly suitable for cases with limited OCT flushing or suspected deep-tissue injury^32,217^. Multimodal imaging further enhances the identification and risk assessment of diseased areas (Fig. 10a(ii)): the OCT-IVUS hybrid system has shown a superior detection rate for SED in comparative clinical analyses versus single-modality systems^221^; the IVUS-NIRS component can comprehensively assess the structural integrity and high-risk plaque areas at the stent edge^222^; and OCT-NIRF imaging can identify edge injuries and microthrombi with high biological activity^223^. Imaging indicates that if the visible external elastic membrane angle is <180° or plaque burden is >50%, it suggests inadequate edge coverage. If a dissection arc is >60°, length >2 mm, involves the media/adventitia, or is flow-limiting, re-dilation or extended stent coverage should be performed. In contrast, superficial, short SEDs with no flow abnormalities can be managed conservatively with imaging follow-up^31,195,208^.

Following cerebral intervention, it is also critical to be vigilant for stent extension or over-coverage that may cause occlusion of perforators or collateral vessels and distal perfusion deficits. While DSA remains the gold standard for cerebral intervention, its resolution is insufficient to fully assess underlying arterial pathologies and therapeutic devices. This is driving the development of miniaturized, high-resolution imaging technologies, such as using photon-counting detector computed tomography angiography (PCD-CTA) with spectral reconstructions to evaluate the peri-stent vascular status^224^. For long-segment peripheral disease, IVUS is frequently employed to delineate the longitudinal and transverse extent of edge dissections involving the media or adventitia^206^.

The identification of tissue protrusion and thrombus is key to preventing acute occlusion during the early post-procedural period. In this regard, OCT can resolve the volume of tissue or thrombus protruding into the lumen and distinguish between red and white thrombi, providing an imaging basis for antithrombotic strategy. When the protrusion or thrombus significantly occupies the effective lumen, additional support or re-dilation should be considered^225–227^. In cerebral vessels, where the caliber is smaller, the wall thinner, and blood flow slower, even minimal tissue protrusion or thrombus can significantly increase the risk of acute stroke or re-occlusion. OCT has been proven highly reliable for imaging thrombus formation on stent surfaces, especially at the ostia of trapped side branches. The emergence of new technologies like HF-OCT has addressed many limitations of existing OCT, such as nearly eliminating non-uniform rotational distortion (NURD) in tortuous paths and significantly expanding the field of view, enabling precise assessment and guidance for early intervention^228,229^.

Furthermore, long-segment calcified lesions, such as those in the lower limbs where calcific rings are thick and extensive, are prone to stent underexpansion and residual calcium plates. This impacts apposition, flow restoration, and long-term patency^230,231^. The role of OCT and IVUS imagings prior to stenting is crucial: repeated imaging is necessary to confirm the fracture of the calcium ring, ensuring the subsequent stent can fully expand. OCT shows a clear advantage in identifying the morphology and depth of these calcium fractures^205,232^.

### Long-term prognostic management and intelligent sensor monitoring

Long-term follow-up assessment requires a comprehensive perspective centered on the three equilibria, vascular mechanical, cellular, and physicochemical-immune ecology^7^, which relies on periodic IVI to monitor device-vessel interactions (Table 5). Neointimal coverage is a key indicator of stent bio-integration. Serial OCT follow-ups can quantify neointimal thickness, coverage, and uniformity to assess healing adequacy. Multiple clinical studies show that the proportion of uncovered struts is highly correlated with late thrombotic events; an OCT-guided follow-up group showed a significantly lower proportion of uncovered struts at 6 months (4.3% vs. 9.0%; *P* < 0.01)^233–235^. HF-OCT can evaluate homogeneous neck coverage by intrasaccular devices and predict successful aneurysm occlusion^214^, as shown in Fig. 10a(iv). Polarization-Sensitive OFDI (PS-OFDI) and related technologies measure polarization changes induced by the birefringence of fibrous tissue, serving as a surrogate for collagen and smooth muscle cell content. Advanced reconstruction algorithms can further extract optical axis metrics of birefringence, elucidating lesion severity and the progression of the healing process. Furthermore, the integration of PS-OFDI with Intravascular Polarimetry (IVP) can additionally indicate the presence of lipid-rich plaque material and macrophages^236,237^. Collectively, these modalities offer novel insights into the vascular healing response following stent implantation.

#### Surveillance of in-stent neoatherosclerosis

Disruption of in-stent neoatherosclerosis is implicated as a critical factor in the pathogenesis of very late stent thrombosis^238^. In the surveillance of neoatherosclerosis, OCT leverages superior spatial resolution to visualize thin fibrous caps and assess microstructural details, yielding higher detection rates for neointimal rupture and thrombosis compared to grayscale IVUS. However, a notable limitation is that large calcified lesions may be misdiagnosed as TCFAs due to the signal attenuation and shadowing inherent to OCT’s limited penetration depth^239^. Conversely, multimodal technologies such as OCT-NIRF or IVUS-NIRF can simultaneously delineate pathophysiological characteristics, including lipid accumulation, endothelial disruption, neovascularization, and zones of macrophage infiltration, holding particular utility for the early identification of high-risk neointima^223,240^.

#### Management of late-acquired malapposition (LAM)

The detection of Late-Acquired Malapposition (LAM) constitutes a critical focal point in longitudinal follow-up. Significant late malapposition is closely associated with an increased risk of very late stent thrombosis^241,242^. Predictors of LAM encompass plaque or thrombus absorption and positive vascular remodeling^242,243^.

While both serial OCT and IVUS are capable of detecting volumetric changes in lumen area and the external elastic membrane, OCT demonstrates superiority over IVUS in detecting subtle intraluminal voids. Management strategies vary by severity: for extensive LAM or cases accompanied by flow abnormalities, selective post-dilation or intensified antiplatelet therapy may be warranted; conversely, conservative observation suffices for the majority of mild LAM cases^196,242,244,245^.

Furthermore, coupling OCT with hemodynamic assessment (e.g., 4D-OCT/4D-flow) enables the analysis of flow disturbances and their impact on late malapposition^246^. Concurrently, photoacoustic imaging has demonstrated efficacy in animal models for detecting vessel wall inflammation and tissue absorption processes^247^. Furthermore, follow-up can be extended to the systematic assessment of non-target lesion progression. By comparing the optical characteristics of native vessels proximal or distal to the stent, such as plaque burden, lipid core size, and fibrous cap thickness, unstable transitions in systemic atherosclerosis can be identified early.

#### Prognostic value of IVUS in non-culprit lesions

The PROSPECT study substantiated that in non-culprit lesions, specific IVUS-derived parameters, namely a plaque burden ≥70%, a minimum lumen area (MLA) ≤ 4 mm², and the presence of TCFAs identified by VH-IVUS, are significantly correlated with future MACE^248,249^. Furthermore, IVUS-based lesion-specific FFR algorithms, which estimate local pressure gradients, enhance the predictive stratification of potential high-risk non-culprit lesions^250^.

#### Surveillance of bioresorbable scaffolds

Regarding scaffold continuity, OCT serves as the gold standard for monitoring bioresorbable scaffolds. In longitudinal follow-up, it effectively identifies structural anomalies such as late discontinuities, partial dismantling, and scaffold collapse. This capability allows for the assessment of structural stability and vascular remodeling status during the resorption process, providing a critical rationale for individualized follow-up and intervention strategies^251,252^.

#### Emerging wearable optical sensors for postoperative care

Wearable sensors introduced in this section represent a non-invasive approach for continuous, real-time postoperative monitoring based on optical technologies. By measuring physiological metrics such as Pulse Wave Velocity (PWV), heart rate, and oxygen saturation (SpO_2_), these devices, when combined with advanced analytics, can assess arterial stiffness, vascular compliance, perfusion status, and endothelial function recovery, thereby enabling the early detection of vascular anomalies^253^.

Photoplethysmography (PPG) is a widely used optical monitoring technique that records light transmission or reflection changes caused by the cardiac cycle. Pulse wave analysis derived from PPG signals reflects heart rate variability, offering insights into cardiovascular health. Depending on the optical path, wearable PPG sensors utilize either transmissive (Fig. 10b(i)) or reflective configurations (Fig. 10b(ii)). While the transmissive mode relies on light penetrating the tissue to a receiver on the opposite side, yielding a higher signal-to-noise ratio, the reflective mode captures backscattered light from superficial tissues, rendering it more suitable for long-term, skin-integrated monitoring^253–256^.

#### Photoacoustic and NIRS innovations

Flexible Photoacoustic Stethoscope: Jin et al.^257^ reported a skin-mountable device capable of continuous, non-invasive, in situ monitoring of multiple cardiovascular biomarkers, including hypoxia, exogenous drug attenuation, and hemodynamics, visualized via customized 3D algorithms (Fig. 10b(iii)).

Near-Infrared Spectroscopy (NIRS): Leveraging the deep tissue penetration of NIR light, NIRS is routinely employed to monitor regional cerebral oxygen saturation (rScO₂) and hemodynamics^258^. Functional NIRS (fNIRS) specifically measures hemodynamic changes via scalp-placed sources and detectors^259^. Ji et al.^260^ engineered a flexible, wearable fNIRS system that offers a lightweight alternative to traditional bulky apparatuses, enabling long-term continuous monitoring of cerebral oxygen supply (Fig. 10b(iv)).

Laser Doppler for peripheral artery disease: For patients with peripheral artery disease, Awan et al.^261^ developed a miniature flowmeter utilizing the laser Doppler effect. This device provides continuous monitoring of blood flow changes in stenotic peripheral arteries, delivering quantitative data critical for early clinical decision-making (Fig. 10b(v)).

To look ahead, wearable vascular monitoring systems are poised to evolve towards greater intelligence, miniaturization, and multimodal integration, propelling the remote dynamic management of PVDs to a superior level of sophistication.

## Conclusions and outlook

This review has summarized four primary imaging modalities centered on optical and acoustic principles (OCT, IVUS, NIRS, and Angioscopy), focusing on their physical mechanisms and system design, and discussing their latest advancements and trends in clinical translation, intelligent integration, and multimodal fusion. Traditional precision intervention has largely focused on device positioning and lesion repair at the time of the procedure, representing what is essentially an instantaneous optimum at a static, spatial level^195,262–265^. Here, we adopt the Imaging-Guided Suitcordance framework in the context of panvascular imaging, which integrates optical imaging throughout the entire workflow of pre-procedural planning, intra-procedural navigation, and post-procedural follow-up. Within this framework, the reviewed evidence suggests that imaging-guided analysis may help move assessment of device-vessel matching from largely static measurements toward more continuous, data-driven evaluation during and after the procedure. The combination of advanced optical catheters and AI-based data analysis is a central thread of this review. Future work is unlikely to be limited to incremental improvements in imaging metrics; it is more likely to involve a gradual evolution of intravascular optical imaging from structural visualization toward intelligent analysis and, in selected settings, integrated theranostic applications.

### Imaging-guided Suitcordance as an evaluation framework

**Fig. 11:**
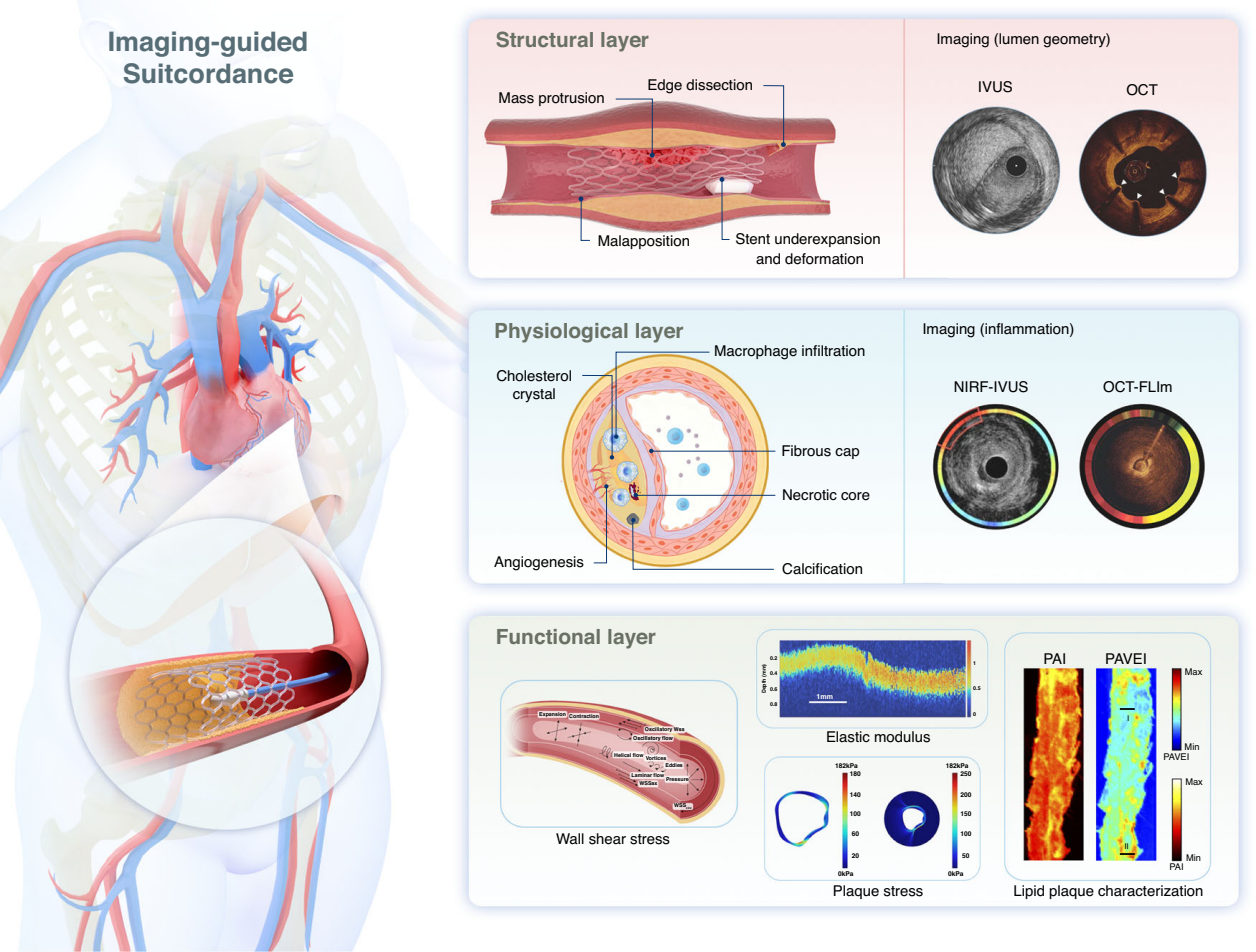
Authors’ conceptual synthesis of Imaging-Guided Suitcordance, informed by the cited literature^10,336^. Three hierarchical levels are delineated. (i) The structural level reflects implantation quality through IVI-based identification of post-procedural anomalies (stent underexpansion, deformation, malapposition, edge dissection, tissue prolapse). (ii) The functional level captures the mechanical balance of the vessel wall via biomechanical readouts (plaque stress, viscoelasticity, elastic modulus, wall shear stress). (iii) The physiological level addresses cellular, molecular and inflammatory responses, covering preoperative plaque composition (fibrous-cap thickness, cholesterol crystals, calcification, macrophage infiltration, necrotic core, neovascularization) and postoperative neoatherosclerosis surveillance^31,115,240,267,276,337–339^. (reproduced with permission from ref. ^115^, ©2025 Kim et al., ref. ^337^, ©2021 Andrea et al., both licensed under CC BY 4.0; ref. ^31^, ©2022 Elsevier; ref. ^240^, ©2024 Springer Nature; ref. ^267^, ©2020 Optica Publishing Group; ref. ^276^, ©2017 Springer Nature; ref. ^338^, ©2019 Wiley; ref. ^339^, ©2024 Wiley;)

Intravascular optical imaging is advancing from “seeing clearly” (structural detail) to “seeing thoroughly” (functional insight), shifting from superficial structural detection (e.g., stent apposition, expansion, residual stenosis, and intraluminal protrusions) to visualization at the functional and molecular levels. To better summarize this evaluation framework, a schematic overview summarizing the structural, functional, and physiological domains is presented in Fig. 11.

Functional imaging focuses on revealing the mechanical ecological balance of the vessel wall, providing a physical basis for predicting plaque rupture and assessing device-vessel mechanical matching by quantitatively evaluating its elastic modulus, shear stress, plaque viscoelasticity and local strain fields^266–272^. For example, OCE, with its high resolution and sensitivity, can quantify the mechanical structure of tissues by mapping their mechanical properties^273–276^. In clinical practice, OCE can be used to quantify plaque stiffness for risk stratification. Plaques with abnormally elevated local stress, even if not causing severe stenosis, should be considered independent risk factors requiring more aggressive intervention and closer follow-up. Huang et al.^277^ proposed an OCT-based method for in vivo biomechanical plaque assessment: by automatically reconstructing plaque structure and incorporating patient-specific pressure data, 2D finite element analysis is used to calculate plaque structural stress (APSS) during the cardiac cycle. The results showed that APSS was significantly elevated in the lesion segment, with peaks often located at the proximal plaque shoulder and minimal lumen area, correlating highly with rupture-prone regions and validating the potential of opto-mechanical modeling in risk prediction. Meanwhile, Photoacoustic Viscoelastic Imaging (PAVEI) technology, used to measure the viscoelastic ratio of atherosclerotic plaques, can enable the identification and warning of vulnerable plaques^278^. Concurrently, molecular imaging technologies will enable the real-time observation of cellular and physicochemical-immune ecological balance. Tracking processes from inflammatory response to matrix remodeling and neointimal healing will provide quantifiable indicators for long-term concordance. For instance, Rau et al.^240^ combined indocyanine green (ICG)-enhanced NIRF with IVUS to identify different stages of atherosclerosis in ex vivo human coronary arteries, finding ICG deposition significantly correlated with macrophage burden and quantifying inflammatory activity. Schneider et al.^279^ utilized near-infrared autofluorescence photoacoustic imaging with ultrasound to achieve high-precision identification of vulnerable plaques by detecting heme degradation products and inflammatory cell signals. In recent years, FLIM-OCT systems have been able to reflect the metabolic state of the vessel wall, such as macrophage infiltration, calcification, and plaque healing, by measuring corresponding tissue fluorescence lifetimes^115,280^. Bec et al.^281^ were the first to integrate FLIM with PS-OCT in a single catheter, synchronously acquiring morphological, birefringent, and biochemical information in vivo without exogenous contrast agents, providing an integrated tool for multi-dimensional assessment of vascular remodeling and inflammation. Note that advanced manufacturing (especially multi-degree-of-freedom schemes) is one cornerstone to support above photonic or multi-model integration and their higher-level miniaturization especially for intravascular spaces. This extension from structural information to biological function is not merely an enhancement of resolution but a cognitive leap in dimensionality—it transforms imaging from a “diagnostic tool” into a “physiological map.”

### Key barriers to translation

On the path from bench to bedside, IVI remains constrained at the hardware, computational and clinical-operations levels. At the hardware level, an unavoidable trade-off exists between spatial resolution and penetration depth, and no single modality can simultaneously deliver high-resolution intraluminal imaging and deep vessel-wall assessment; to date, no single imaging technique is capable of a fully comprehensive plaque evaluation^108^. Although multimodal fusion mitigates this limitation by combining complementary modalities, it does so at the expense of overall system stability. In complex vascular anatomies, motion artifacts, signal attenuation and mis-registration continue to compromise the reliability of in vivo imaging^222^. In tortuous and small-caliber vessels, IVI must simultaneously preserve imaging fidelity and mechanical trackability, imposing stringent constraints on catheter miniaturization. At the computational level, the principal bottlenecks are real-time data throughput and the stability of image segmentation: under the concurrent demands of high-throughput intra-procedural acquisition and low-latency decision support, current systems still fall short of true clinical real-time performance. AI-based segmentation is prone to failure when faced with calcification- or lipid-induced signal attenuation and diverse artefacts, and it still exhibits clear shortcomings in recognizing safety-critical qualitative features, for example, stent-edge dissection and the discrimination of thrombus from artifact, thereby weakening its diagnostic value. Moreover, available training datasets remain limited, and because the ultimate therapeutic decision relies on the clinician’s holistic judgment together with patient-specific factors, current AI systems cannot yet replace the expert experience required for IVI-guided PCI^282^. At the clinical-operations level, procedural complexity, longer operation times, more contrast use and operator variability restrict routine adoption of IVI^283^; simultaneously, lengthy regulatory-approval and clinical-validation pathways further delay the translation of AI-assisted IVI systems into practice. For some emerging systems, large-scale trials on hybrid IVI guiding pharmacological or interventional therapy are still needed to confirm efficacy and establish clinical value^222^.

### Future directions

The convergence of AI and digital twins is expected to shift IVI from “recognition support” to “predictive decision-making”. Future development will focus on digital twin-based virtual intervention systems that integrate patient-specific anatomy, physiological parameters, and multi-physics modeling, enabling simulation of procedural outcomes and improving prediction of therapeutic effects and decision optimization. However, constructing a digital twin depends on high-quality, comprehensive data, and data insufficiency directly limits the model’s individualization and accuracy, thereby eroding clinical utility. Obstacles on the data side, difficulty of acquisition, quality heterogeneity and the challenges of multi-source fusion, jointly lower model reliability and precision. In addition, limited interpretability, high computational cost and poor integration with clinical workflows further impede adoption. Most existing studies remain at the preclinical or proof-of-concept stage, with a paucity of high-quality randomized controlled trials and multi-center validation, which collectively restrains the overall pace of clinical translation^284^.

To facilitate clinical translation, future efforts should prioritize phased validation in controlled settings such as coronary interventions, followed by extension to peripheral and cerebrovascular applications. At the same time, integrating multimodal IVI data into standardized, interoperable platforms, together with prospective multicentre validation and regulatory-grade evaluation of AI-enabled digital twin systems, will be essential to ensure clinical translation. Such advances may help bridge proof-of-concept systems and clinically deployable digital twins.

Finally, the deep integration of optical imaging with therapeutic modalities may drive the evolution of a theranostic closed loop. Advances in intravascular optical imaging, particularly AO-IVUS, combined with MEMS-based miniaturization and multimodal catheter probes, are enabling the integration of diagnosis, feedback, and localized therapy within a single device platform^45,101,222^. Future progress will rely on extending intravascular photonic systems from imaging toward adaptive theranostic applications through improved control of laser-tissue interactions and integrated device functionality. However, major challenges remain, including the persistent trade-off between imaging resolution and penetration depth, instability of the optical-biological interface under blood-scattering conditions, latency within real-time optical feedback loops, and the absence of reliable predictive models for heterogeneous plaque microenvironments. Catheter-scale integration is further constrained by multimodal miniaturization, optical alignment tolerances, energy delivery and thermal safety.

To realize clinically deployable theranostic closed loop systems, future development should move beyond technology aggregation toward phased translational implementation, including staged validation in controlled coronary settings followed by extension to peripheral and cerebrovascular applications, integration of multimodal IVI data into standardized platforms. Regulatory-grade validation of AI components will also be required. Progressive deployment of robot-assisted imaging-guided workflows may then bridge prototype systems and routine clinical practice. Beyond phased translational deployment, future clinically executable closed-loop systems may increasingly depend on a robust “AI + photonics” framework. Specifically, deep learning architectures can be leveraged to overcome classical optical diffraction limits, enabling computational super-resolution that reveals structural details far beyond the reach of standard hardware. Moreover, to address the critical bottlenecks of real-time processing and massive data throughput, the deployment of edge AI chips directly at the catheter distal tip offers a transformative solution. By performing on-site photon feature extraction, these intelligent circuits significantly reduce transmission bandwidth requirements and latency, facilitating a truly seamless, real-time navigation and guidance system for complex panvascular interventions.

In summary, the future of panvascular intervention should focus on clinically executable closed-loop systems rather than isolated technological advances. The key challenge lies in translating computational photonics and AI-based modeling into stable, workflow-compatible clinical systems. Within this context, Suitcordance provides an integrative framework. By linking digital vascular models with interventional decision-making, it enables therapies that are more precise and dynamically responsive to the evolving vascular microenvironment.
